# Antimicrobial susceptibility and virulence traits of *Escherichia coli* in healthy South American camelids in Germany

**DOI:** 10.3389/fmicb.2026.1898372

**Published:** 2026-07-21

**Authors:** Aung Zaw Moe, Carsten Heydel, Henrik Wagner, Lisa Ulrich, Torsten Semmler, Christian Berens, Christa Ewers

**Affiliations:** 1 Institute of Hygiene and Infectious Diseases of Animals, Faculty of Veterinary Medicine, Justus Liebig University, Giessen, Germany; 2 Veterinary Clinic for Reproductive Medicine and Neonatology, Justus Liebig University, Giessen, Germany; 3 Genomic Surveillance Unit, Robert Koch Institute, Berlin, Germany; 4 Friedrich-Loeffler-Institut, Institute of Molecular Pathogenesis, Jena, Germany

**Keywords:** alpaca, antimicrobial susceptibility, *Escherichia coli*, llama, pathotype, sequence type, whole-genome sequencing

## Abstract

South American camelids (SACs) are gaining popularity in the leisure sector, leading to human contact. However, data on pathogenic, zoonotic, and antimicrobial-resistant (AMR) bacteria in SACs remain limited. This study investigated the occurrence of AMR and virulence determinants in *Escherichia coli* isolated from feces of healthy alpacas and llamas in Germany. Between April 2021 and February 2023, longitudinal sampling was conducted on up to 20 animals per farm from 10 farms across six German federal states, with four sampling rounds at 6-month intervals. One random *E. coli* isolate per sample was obtained using a non-selective approach and tested for susceptibility to 14 antibiotics by broth microdilution, following EUCAST guidelines. In parallel, selective screening was performed to detect colistin- and carbapenem-resistant strains as well as ESBL/AmpC *β*-lactamase producers. Whole-genome sequence data from non-selectively obtained isolates mainly from round A and selectively obtained isolates from all rounds were used to identify AMR determinants and virulence-associated markers. Overall, 706 non-selectively isolated *E. coli* showed low resistance rates: sulfamethoxazole (4.5%), trimethoprim (1.8%), tetracycline (1.6%), ampicillin (1.4%), enrofloxacin (0.4%), cefotaxime (0.4%), colistin (0.1%), amoxicillin/clavulanate (0.1%), and piperacillin/tazobactam (0.1%). Eleven isolates exhibited multidrug-non-wild-type (MD-non-WT) phenotypes to ≥3 antimicrobial classes. Selective screening identified 18 *mcr-1* and 70 cefotaxime non-WT isolates, but no carbapenem non-WT isolates. Resistance genes included *bla*_CTX-M-1/14/15/27/55_ (*n =* 54), *bla*_DHA-1_ (*n =* 4), *bla*_CMY-2_ (*n =* 4) and *ampC* promoter mutations (C42T) (*n =* 2). All colistin-resistant isolates carried *mcr-1.1* on an IncI2 plasmid and originated from a single farm. Multilocus VNTR analysis revealed high genetic diversity with farm-specific clustering and temporal shifts. Although overall AMR prevalence was low, localized presence of ESBL- and AmpC-*β*-lactamase-producing and colistin-resistant *E. coli* indicates that SACs may represent AMR sources. Virulence profiling revealed few pathogenic *E. coli* except Shiga toxin-producing *E. coli*. These included low-virulence *stx* subtypes (*stx1c*, *stx2b*, and *stx2f*) and the high-virulence subtype *stx2d*, associated with enterohemorrhagic *E. coli* (EHEC) causing hemorrhagic colitis and hemolytic uremic syndrome in humans. Notably, detection of an EHEC isolate belonging to the O63:H6/ST583 lineage, a serotype/sequence type previously associated with severe clinical outcomes, highlights a zoonotic risk associated with SACs.

## 1 Introduction

South American camelids (SACs), including guanacos (*Lama guanicoe*), vicuñas (*Vicugna vicugna*), llamas (*Lama glama*), and alpacas (*Vicugna pacos*), are increasingly kept in non-native regions such as Germany, primarily as companion animals or for leisure activities such as trekking (Zarrin et al., 2020). Their growing proximity to humans raises concerns about their potential role in the transmission of zoonotic pathogens, including antimicrobial-resistant (AMR) bacteria. Previous studies have identified a range of zoonotic agents in SACs, suggesting that these animals may serve as reservoirs for pathogens with public health relevance (González-Santamarina et al., 2022a; Konieczny and Pomorska-Mól, 2024; Menge et al., 2026; Moe et al., 2026). However, little is known about the role of SACs as reservoirs of antimicrobial-resistant and potentially pathogenic *E. coli*.

*Escherichia coli* is a key indicator organism for assessing AMR dissemination and zoonotic risk at the animal-human-environment interface. Due to its ubiquity and genetic plasticity, this species readily acquires resistance determinants through horizontal gene transfer and is therefore widely used as a sentinel for AMR surveillance (EFSA, 2025). Of particular concern are multidrug-resistant (MDR) *E. coli* carrying extended-spectrum *β*-lactamases (ESBL), plasmid-mediated AmpC β-lactamases (AmpC), carbapenemases (CP), or mobile colistin resistance genes (*mcr*) (Bush and Bradford, 2020; Ewers et al., 2022). Accordingly, *E. coli* is designated as an indicator organism for AMR monitoring in food-producing animals and environmental reservoirs under EU legislation (Commission Implementing Decision, 2020), enabling harmonized, cross-sectoral comparison of resistance trends (EFSA, 2025). However, SACs are currently not included in the EU AMR surveillance programs, despite indications that they may represent an overlooked reservoir of resistant *E. coli* strains (González-Santamarina et al., 2022a; Dost et al., 2023).

Beyond antimicrobial resistance, the pathogenic potential of *E. coli* strains circulating in SACs also remains poorly understood. The bacterium is a natural component of the intestinal microbiota of SACs (González-Santamarina et al., 2022a), as in most animal species, but also comprises pathogenic variants capable of causing intestinal and extraintestinal disease (Mailyan, 2016). In SACs, *E. coli* infections have been associated with a range of clinical conditions, including diarrhea, metritis, mastitis, meningitis, and septicemia (Staples, 2016). Pathogenic *E. coli* are broadly classified into distinct intestinal pathogenic *E. coli* (InPEC) and extraintestinal pathogenic *E. coli* (ExPEC) based on their virulence mechanisms and clinical manifestations. Among InPEC, Shiga toxin-producing *E. coli* (STEC), including enterohemorrhagic *E. coli* (EHEC), are of major public health relevance due to their ability to cause severe disease in humans, such as hemorrhagic colitis and hemolytic uremic syndrome (Karmali, 2017). Ruminants are recognized as major reservoirs of STEC, facilitating zoonotic transmission via food and environmental contamination (van Hoek et al., 2019). ExPEC, including uropathogenic (UPEC) and sepsis-associated *E. coli* (SEPEC), are implicated in a range of extraintestinal infections and may originate from the intestinal tract of healthy animals (Bélanger et al., 2011).

Despite the relevance of *E. coli* as both an AMR indicator and a zoonotic pathogen, comprehensive genomic data on isolates from SACs – particularly regarding phylogeny, virulence potential, and resistance profiles remain scarce. To address this knowledge gap, the present study applies whole-genome sequencing to characterize SAC-derived isolates in Germany to evaluate their potential role as reservoirs of antimicrobial resistance and zoonotic virulence traits.

## 2 Materials and methods

### 2.1 Animal sampling

As part of the joint project “Development and establishment of a multi-stage animal health management for farms with South American camelids (SACs),” ten SAC farms across Germany were included in this study. Farms were distributed across six federal states. Up to 20 animals per farm were sampled during four visits conducted at 6-month intervals between April 2021 and February 2023. For data protection reasons, the names of farms and federal states are not disclosed.

Five farms (nos. 4, 6, 7, 9, and 10) exclusively kept alpacas, one farm (no. 5) only llamas, while the remaining farms (nos. 1, 2, 3, and 8) housed both species. One farm (farm no. 9) participated only in the first sampling round. Some farms also kept additional animals either on-site or in close proximity. Farms 1–3 maintained diverse animal holdings, including chickens, sheep, goats, kangaroos, peacocks, reindeer, ducks, horses, Old World camels, guanacos, donkeys, and parrots. Farm 4 kept cattle on-site and horses nearby, while farms 6 and 7 had horses in the vicinity. No additional animal species were reported on farms 5, 8, 9, or 10.

Approximately 25 g of fecal samples were collected directly from the rectum using gloved hands and transferred into sterile 100 mL containers. Samples were transported to the laboratory within 24 h and processed immediately.

For bacterial enrichment, fecal material was incubated overnight in peptone water at 37 °C and subsequently stored frozen in brain heart infusion (BHI) broth (Oxoid, CM1135) supplemented with 30% glycerol.

All procedures were conducted in accordance with applicable animal welfare regulations and were approved by the District Council of Giessen (approval number: V 54–19 c 20 15 h 02 GI 18/4 Nr. A 3/2021).

### 2.2 Isolation of *E. coli*

For non-selective (non-SEL) isolation, enriched fecal suspensions were rapidly thawed at 50 °C for 2–3 min (Ternent et al., 2004), and 10 μL were streaked onto MacConkey agar plates (Oxoid, UK) without antimicrobials. After incubation at 37 °C for 24 h, one colony morphologically consistent with *E. coli* was subcultured on fresh MacConkey agar plates and incubated for an additional 24 h at 37 °C.

Selective cultivation (SEL) was performed in parallel to recover presumptive non-wild-type (non-WT) isolates with respect to selected antimicrobial agents. Samples were streaked onto five selective media: MacConkey agar supplemented with tigecycline (1 μg/mL), cefotaxime (1 μg/mL), or amikacin (16 μg/mL), as well as CHROMagar™ mSuperCARBA™ and CHROMagar™ COL-APSE (Commission Implementing Decision, 2020; EFSA, 2024). Plates were incubated at 37 °C for 48 h.

Isolates obtained on antimicrobial-containing media were further analyzed to confirm non-WT phenotypes or detect relevant antimicrobial-resistance determinants. Isolates recovered from colistin-containing medium were screened by PCR for *mcr* genes (*mcr*-1 to *mcr*-10) (Göpel et al., 2024b). Isolates recovered from amikacin- and cefotaxime-containing media were tested for antimicrobial susceptibility using the EUCAST disk diffusion method (version 12.0) (EUCAST, 2024). Inhibition zone diameters were interpreted using EUCAST epidemiological cutoff values (ECOFFs) obtained from the EUCAST database to classify isolates as wild-type (WT) or non-wild-type (non-WT) (EUCAST, 2023). Presumptive tigecycline non-WT isolates were whole-genome sequenced to screen for *tet*(X) genes. From each selective medium, one presumptive *E. coli* colony was stored.

Isolates were confirmed as *E. coli* by matrix-assisted laser desorption/ionization time-of-flight mass spectrometry (MALDI-TOF MS; Biotyper microflex LT/SH, library v.10.0.0.0, Bruker Daltonics, Bremen, Germany). Only isolates with an identification score ≥2.0 were included (Ahmed et al., 2021) and stored in BHI medium (Oxoid, CM1136) with 30% glycerol at −70 °C.

### 2.3 Antimicrobial susceptibility testing

Antimicrobial susceptibility testing (AST) of non-selectively obtained *E. coli* isolates (*n =* 706) was performed using the broth microdilution method (Micronaut-S system, v6.00; Bruker Daltonics) in accordance with EUCAST guidelines (EUCAST, 2023). A customized microtiter plate layout (E1-301-100) based on the specifications of the German zoonoses monitoring program was used. The panel included 14 antimicrobial agents or combinations at the following concentration ranges (μg/mL): amikacin (0.25–32), amoxicillin/clavulanic acid (1/0.5–16/8), ampicillin (1–16), cefotaxime (0.016–2), ceftazidime (0.031–8), colistin (0.125–8), enrofloxacin (0.004–2), florfenicol (1–16), gentamicin (0.125–8), piperacillin/tazobactam (0.5/4–64/4), spectinomycin (4–64), sulfamethoxazole (4–256), tetracycline (0.25–8), and trimethoprim (0.25–8) (Richter et al., 2025).

For testing, isolates were cultured on 5% sheep blood agar (Merck, Darmstadt, Germany), and single colonies were used to prepare a bacterial suspension (0.5 McFarland) in 0.9% NaCl (Merck). The suspension was transferred into cation-adjusted Mueller-Hinton broth (MERLIN Diagnostika GmbH, Bornheim-Hersel, Germany) and used to inoculate microtiter plates according to the manufacturer’s instructions.

After incubation at 37 °C for 18–20 h, minimum inhibitory concentrations (MICs) were determined using a microplate reader (Thermo Fisher Scientific, Vantaa, Finland) with MCN6 software (MERLIN Diagnostika) or visually, as recommended for sulfamethoxazole and trimethoprim (CLSI, 2012). Quality control included the reference strains *E. coli* ATCC 25922 and NCTC 13846.

AST results were interpreted using epidemiological cutoff values (ECOFFs), and isolates were classified as WT or non-WT accordingly (EUCAST, 2023). Sulfamethoxazole interpretation followed Commission Implementing Decision (EU) 2020/1729 (Commission Implementing Decision, 2020). Because susceptibility interpretation in the present study was based on epidemiological cutoff values (ECOFFs) rather than clinical breakpoints, the term multidrug-resistant (MDR) was not used. In addition, the absence of host-specific clinical breakpoints for SACs further precluded formal MDR classification (Sweeney et al., 2018). Therefore, isolates showing non-WT phenotypes to at least one antimicrobial agent in three or more antimicrobial classes were classified as multidrug-non-WT (MD-non-WT), based on the MDR framework proposed by Magiorakos et al. (2012), although without clinical resistance interpretation.

### 2.4 Whole-genome sequencing

Genomic DNA was extracted using the MasterPure DNA Purification Kit (Epicentre Biotechnologies, Madison, WI, USA) according to the manufacturer’s instructions. Library preparation was performed using the Nextera XT DNA Library Preparation Kit (Illumina Inc., San Diego, CA, USA). Whole-genome sequencing (WGS) was conducted on an Illumina MiSeq platform generating 250-bp paired-end reads. Purified DNA from a subset of the isolates was sent to Novogene (Martinsried, Germany) for whole-genome sequencing (Illumina NovaSeq 6000 or NovaSeq X Plus, 150-bp paired-end reads). Sequencing resulted in an average genome coverage exceeding 50× per isolate, providing sufficient depth for reliable genome assembly and downstream analyses.

Raw reads were quality-trimmed and adapter-filtered using Flexbar v3.0.3, followed by quality assessment with FastQC v0.11.7 and MultiQC v1.6. Adapter-trimmed reads were assembled *de novo* using SPAdes v3.15.1 with read correction enabled. Scaffolds shorter than 500 bp were discarded. Assembly quality was evaluated using QUAST v5.0.0 with default parameters. Draft assemblies were excluded if the N50 was below 30 kbp or if assemblies contained more than 900 contigs. Genome annotation was performed using the RAST server with the RASTtk pipeline.^1^

### 2.5 *In silico* analysis of *E. coli* genome sequences

#### 2.5.1 Sequence typing and phylogenetic analyses

Multilocus sequence types (STs) were determined using Ridom SeqSphere+ v.10.0.5 (Ridom GmbH, Münster, Germany) according to the Achtman seven-gene MLST scheme comprising the housekeeping genes *adk, fumC, gyrB, icd, mdh, purA*, and *recA* (Wirth et al., 2006). Serotypes were predicted *in silico* using the integrated SeqSphere+ typing tool *E. coli* CGE SerotypeFinder.

Phylogenetic grouping was assigned using the web-based ClermontTyper tool.^2^ This enabled classification into *Escherichia albertii, Escherichia fergusonii, Escherichia* cryptic clades I–V, *E. coli sensu stricto*, and phylogroups A, B1, B2, C, D, E, F, and G (Beghain et al., 2018; Clermont et al., 2019).

For high-resolution phylogenetic analysis and assessment of clonal relationships, core genome MLST (cgMLST) analysis was performed using Ridom SeqSphere+ v.10.0.5. Allelic distances were calculated using the “pairwise ignore missing values” option, and a minimum spanning tree (MST) was generated. Clusters of closely related isolates were defined using a maximum allelic distance threshold of 10 alleles and a minimum cluster size of two isolates (Biguenet et al., 2023).

#### 2.5.2 Detection of antimicrobial resistance determinants and pathotype classification using virulence-associated genes

Antimicrobial resistance (AMR) genes, chromosomal point mutations, and plasmid replicons were identified using Ridom SeqSphere+ v.10.0.5. Detection was performed using the integrated *E. coli* NCBI AMRFinderPlus and Chromosome & Plasmid Overview databases implemented in Ridom SeqSphere+. Gene detection was based on default SeqSphere+ parameters, applying thresholds of >90% nucleotide identity and >90% sequence coverage.

Virulence-associated genes (VAGs) were identified using Ridom SeqSphere+ v.10.0.5 based on the integrated *E. coli* virulence gene database implemented in the software. Pathotype classification was based on the presence of characteristic VAGs as described previously (Johnson et al., 2003; Göpel et al., 2024b). Isolates were classified as intestinal pathogenic *E. coli* (InPEC) or extraintestinal pathogenic *E. coli* (ExPEC) according to established virulence gene criteria (Johnson et al., 2003; Göpel et al., 2024b).

InPEC were defined as isolates carrying at least one of the following virulence determinants: adhesive fimbriae F4 (*faeG*), F5 (*fanA*), F6 (*fasA*), F18 (*fedA*), or F41 (*fimF41a*); enterotoxins LT-Ia, LT-Ib (*eltB-Ip*), ST-Ia (*estap*), or ST-II (*estb*); Shiga toxins (*stx1*, *stx2*); or intimin (*eae*) (Göpel et al., 2024b). InPEC were further subclassified as attaching and effacing *E. coli* (AEEC) when *eae* was present, Shiga toxin-producing *E. coli* (STEC) when *stx1* and/or *stx2* were detected, enterohemorrhagic *E. coli* (EHEC) when both *eae* and *stx1*/*stx2* were present, and enterotoxigenic *E. coli* (ETEC) when genes encoding adhesive fimbriae in combination with heat-labile and/or heat-stable enterotoxin genes were detected (Göpel et al., 2024b).

ExPEC were defined by the presence of at least two of the following five VAGs: *papA*/*papC* (P fimbriae), *sfa/foc* (S and F1C fimbriae), *afa/dra* (Dr-binding adhesins), *iutA* (aerobactin acquisition system), and *kpsMT* II (capsule synthesis) (Johnson et al., 2003). ExPEC pathotypes were further defined as follows: uropathogenic *E. coli* (UPEC), additionally carrying *cnf1* or *hlyD* (Johnson et al., 2005); sepsis-associated *E. coli* (SEPEC), additionally carrying one of the genes *cnf1, cnf2, cdtB,* or *hlyF* (Mitchell et al., 2015; Sarowska et al., 2019).

### 2.6 Multiple-locus variable-number tandem-repeat (VNTR) analysis (MLVA)

MLVA typing was performed to characterize non-SEL *E. coli* isolates using a seven-VNTR-locus protocol as previously described (Caméléna et al., 2019; González-Santamarina et al., 2022a). The VNTR loci analyzed were *rhaD*, *rsxC*, *ftsK*, *tolA*, *ytfL*, tRNA-Arg, and *hemY*.

DNA was extracted using boiling lysates. The primer mix was prepared by combining individual primers at a final concentration of 10 pmol/μL. PCR amplification was carried out in a final reaction volume of 25 μL, consisting of 23 μL of master mix and 2 μL of DNA template. The master mix contained 2.5 μL of 10× DreamTaq Green Buffer (Thermo Fisher Scientific, Waltham, MA, USA), 0.5 μL of 10 mM dNTPs (Rapidozym GmbH, Berlin, Germany), 0.15 μL of DreamTaq DNA Polymerase (Thermo Fisher Scientific), 12.35 μL of sterile deionized water, and 7.5 μL of a primer mix containing all seven VNTR primer pairs (Eurofins Genomics, Ebersberg, Germany).

Thermocycling conditions were as follows: 94 °C for 15 min; 30 cycles of 94 °C for 30 s, 58 °C for 90 s, and 68 °C for 90 s; followed by a final extension at 68 °C for 10 min. Amplified fragments were separated on 2.5% agarose gels (anprotec, Bruckberg, Germany), stained with ethidium bromide, electrophoresed for 1 h at 120 V, and visualized under UV light. Each gel included three lanes loaded with a 100 bp Plus DNA ladder (Thermo Scientific, Villebon-sur-Yvette, France) for normalization. Gel images were analyzed using BioNumerics v.6.6 (Applied Maths, Sint-Martens-Latem, Belgium). Dendrograms were generated using the unweighted pair group method with arithmetic mean (UPGMA) based on the Dice similarity coefficient, with a position tolerance of 1% and an optimization setting of 2%.

## 3 Results

### 3.1 Sample collection

A total of 706 fecal samples were collected, including 423 from alpacas, 281 from llamas, and two from a huarizo (llama–alpaca hybrid). The targeted number of 20 samples per farm and sampling round could not always be achieved due to differences in herd size and animal availability. Consequently, fewer samples were obtained in some sampling rounds, resulting in 194 samples in round A, 175 in round B, 174 in round C, and 163 in round D.

Across farms, the number of samples collected ranged from 69 to 80 per farm, with the exception of farm 9, which contributed only 20 samples as it participated only in sampling round A. The target number of samples was reached only on farm 10.

Because sampling was conducted repeatedly over multiple rounds, some animals were sampled more than once, enabling longitudinal comparison of *E. coli* occurrence over time. In total, 272 animals were sampled once, 94 twice, 46 three times, and 27 four times.

### 3.2 Bacterial isolation and antimicrobial susceptibility testing

#### 3.2.1 Non-selective cultivation approach

*Escherichia coli* was isolated from all 706 fecal samples using the non-selective cultivation approach (non-SEL), resulting in 706 isolates subjected to antimicrobial susceptibility testing (AST).

Overall, 36 isolates (5.1%) exhibited a non-WT phenotype toward at least one antimicrobial agent. Non-WT phenotypes were most frequently observed for sulfamethoxazole (4.5%), followed by trimethoprim (1.8%), tetracycline (1.6%), ampicillin (1.4%), spectinomycin (0.7%), and florfenicol (0.6%) (Table 1). Reduced susceptibility to critically important antimicrobials, including third-generation cephalosporins and colistin, was rare (Table 2). All isolates were classified as WT for amikacin.

**Table 1:** Proportion of non-wild-type isolates among 706 non-selectively obtained *E. coli* isolates.

Antimicrobial	Farm 1 (*n =* 77)	Farm 2 (*n =* 74)	Farm 3 (*n =* 78)	Farm 4 (*n =* 78)	Farm 5 (*n =* 69)	Farm 6 (*n =* 73)	Farm 7 (*n =* 79)	Farm 8 (*n =* 78)	Farm 9 (*n =* 20)	Farm 10 (*n =* 80)	Total (n)	Total (%)
Amikacin	0	0	0	0	0	0	0	0	0	0	0	0.0
Amoxicillin/clavulanic acid	0	0	1	0	0	0	0	0	0	0	1	0.1
Ampicillin	0	3	1	1	1	1	3	0	0	0	10	1.4
Cefotaxime	0	0	1	0	0	0	2	0	0	0	3	0.4
Ceftazidime	0	0	0	0	0	0	2	0	0	0	2	0.3
Colistin	0	0	0	0	0	1	0	0	0	0	1	0.1
Enrofloxacin	0	0	0	0	0	1	2	0	0	0	3	0.4
Florfenicol	0	0	1	0	0	0	3	0	0	0	4	0.6
Gentamicin	0	0	0	0	0	0	2	0	0	0	2	0.3
Piperacillin/tazobactam	0	0	1	0	0	0	0	0	0	0	1	0.1
Spectinomycin	0	0	1	1	0	0	3	0	0	0	5	0.7
Sulfamethoxazole	2	3	4	2	1	3	7	8	0	2	32	4.5
Tetracycline	0	2	1	2	1	2	3	0	0	0	11	1.6
Trimethoprim	0	2	1	2	1	0	4	3	0	0	13	1.8

Values represent the number of antimicrobial non-wild-type (non-WT) *E. coli* isolates per farm based on epidemiological cutoff values (ECOFFs), indicating the presence of acquired resistance mechanisms. Percentages refer to the proportion among all 706 isolates tested.

**Table 2:** Prevalence of non-wild-type *E. coli* isolates and classification of tested antimicrobial agents according to WHO AWaRe and EMA criteria.

Antimicrobial agent	Antimicrobial (sub-) class	Non-wild-type(among 706 isolates)		WHO AWaRe category *	EMA category **
		No.	%		
Spectinomycin	Aminocyclitol	5	0.7	Access	Prudence
Amikacin	Aminoglycoside	0	0.0	Access	Caution
Gentamicin	Aminoglycoside	2	0.3	Access	Caution
Florfenicol	Amphenicol	4	0.6	Not listed	Caution
Amoxicillin/clavulanic acid	Aminopenicillin (beta-lactam) & β-lactamase inhibitor	1	0.1	Access	Caution
Ampicillin	Aminopenicillin (beta-lactam)	10	1.4	Access	Prudence
Cefotaxime	Third generation cephalosporin (beta-lactam)	3	0.4	Watch	Restrict
Ceftazidime	Third generation cephalosporin (beta-lactam)	2	0.3	Watch	Restrict
Piperacillin/tazobactam	Ureidopenicillin (beta-lactam) & β-lactamase inhibitor	1	0.1	Watch	Avoid
Colistin	Polymyxin	1	0.1	Reserve	Restrict
Enrofloxacin	Quinolone	3	0.4	Not listed	Restrict
Sulfamethoxazole	Sulfonamide	32	4.5	Access	Prudence
Trimethoprim	Dihydrofolate reductase inhibitor	13	1.8	Access	Prudence
Tetracycline	Tetracycline	11	1.6	Access	Prudent

*The World Health Organization (WHO) AWaRe (Access, Watch, Reserve) antibiotic category (WHO, 2022). **European Medicines Agency (EMA) antibiotic category: Avoid – A, Restrict – B, Caution – C, Prudence – D (European Medicines Agency, 2020).

Eleven isolates (1.6%) exhibited non-WT phenotypes to at least three, and up to 10 antimicrobial agents representing up to nine antimicrobial classes (Table 3) and were therefore classified as multidrug-non-WT (MD-non-WT) isolates according to the criteria applied in this study.

**Table 3:** MIC profiles of non-selectively obtained *E. coli* isolates showing non-wild-type phenotypes to ≥ three antimicrobial classes.

Isolate	Antimicrobial agents and antimicrobial susceptibility (MIC, mg/L)													
	AMC	AMK	AMP	CAZ	COL	CTX	ENR	FLL	GEN	PIT	SMO	SPT	TET	TRP
3C49-NS-EC-1	16	1	> 16	0.125	0.5	0.5	0.0156	> 16	0.25	32	>256	> 64	> 8	> 8
7B31-NS-EC-1	4	1	> 16	8	0.25	> 2	> 2	> 16	> 8	1	>256	> 64	> 8	> 8
7D76-NS-EC-1	4	4	> 16	8	0.25	> 2	> 2	> 16	> 8	1	>256	> 64	> 8	> 8
7D61-NS-EC-1	2	2	8	0.25	1	0.0625	0.0313	> 16	0.5	1	>256	> 64	> 8	> 8
2A07-NS-EC-1	4	1	> 16	0.25	0.5	0.0625	0.0313	4	0.5	1	>256	32	> 8	> 8
4C51-NS-EC-1	8	2	> 16	0.125	0.25	0.0313	0.0313	4	0.25	1	>256	32	> 8	> 8
5C59-NS-EC-1	4	1	> 16	0.125	0.25	0.0313	0.125	4	0.25	0.5	>256	32	> 8	> 8
2D79-NS-EC-1	4	1	> 16	0.125	0.5	0.0625	0.0313	4	0.5	1	>256	32	1	> 8
2B22-NS-EC-1	4	4	> 16	0.125	0.5	0.0625	0.0313	4	0.5	1	>256	32	> 8	0.25
6B37-NS-EC-1	2	4	2	0.125	4	0.0313	2	4	1	1	8	32	> 8	0.25
4A07-NS-EC-1	2	2	2	0.125	0.25	0.0625	0.0313	8	0.5	1	16	> 64	> 8	> 8

Non-wild-type (non-WT) MICs, defined as values above the ECOFF, are highlighted in gray. AMC, amoxicillin/clavulanic acid; AMK, amikacin; AMP, ampicillin; CAZ, ceftazidime; COL, colistin; CTX, cefotaxime; ENR, enrofloxacin; FLL, florfenicol; GEN, gentamicin; PIT, piperacillin/tazobactam; SMO, sulfamethoxazole; SPT, spectinomycin; TET, tetracycline; TRP, trimethoprim.

#### 3.2.2 Selective cultivation approach

Selective cultivation (SEL) increased the detection of isolates carrying clinically relevant resistance determinants. A total of 70 cefotaxime non-WT isolates and 18 *mcr*-*1*-positive isolates were identified. All *mcr-1*-positive isolates originated from a single farm and were predominantly (94.7%) detected during sampling round A (Supplementary Table S1).

No *E. coli* isolates were recovered from carbapenem-containing media. Isolates recovered on tigecycline- and amikacin-containing media did not harbor known acquired high-level resistance determinants associated with these phenotypes.

### 3.3 Whole-genome sequencing

Whole-genome sequencing (WGS) was performed for a subset of *E. coli* isolates selected based on sampling round and non-WT phenotype. All 194 *E. coli* isolates obtained from the initial sampling round (round A) through the non-SEL cultivation approach were included in the WGS analysis. In addition, four isolates from other sampling rounds were selected due to non-WT phenotypes toward colistin and third-generation cephalosporins, as determined by broth microdilution testing. In total, 198 non-selectively obtained isolates were subjected to WGS.

From the selective cultivation approach, 116 isolates were subjected to WGS. These included 18 *mcr-1*-positive isolates, 70 non-WT isolates to cefotaxime, 13 non-WT isolates to amikacin, and 15 isolates recovered from tigecycline-containing media.

### 3.4 Multilocus sequence types (STs) and phylogenetic grouping

#### 3.4.1 Non-selectively obtained isolates

**Figure 1:**
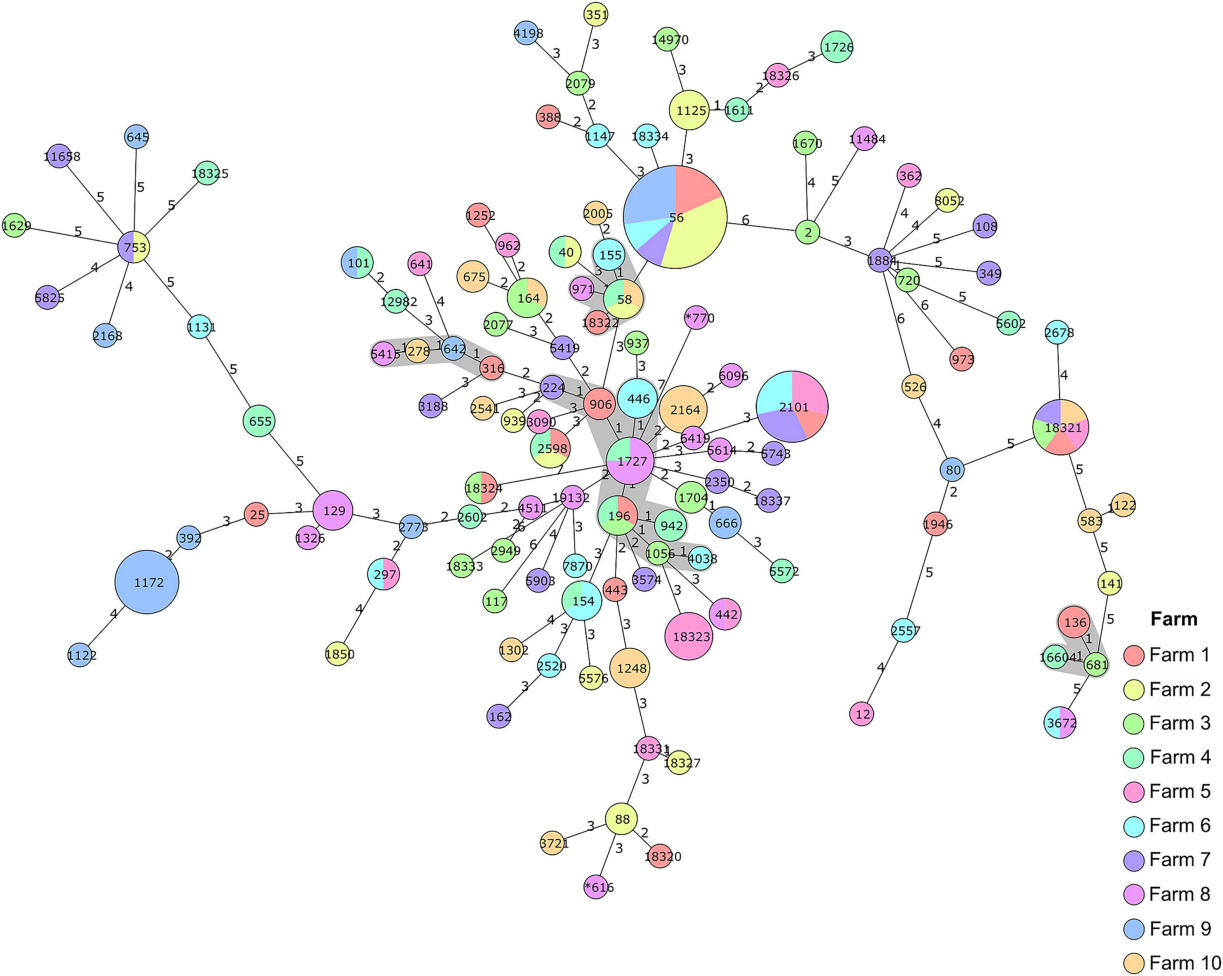
Minimum spanning tree (MST) of 194 non-selectively obtained *E. coli* isolates from sampling round A, based on the multilocus sequence typing (MLST) alleles. Each node represents a unique sequence type (ST), and node size is proportional to the number of isolates assigned to that ST. Numbers on the connecting lines indicate allelic differences across the seven MLST loci. Colors indicate the farm origin.

MLST analysis of the 198 non-selectively obtained isolates, comprising 194 isolates from sampling round A and four isolates from other sampling rounds (B, C, and D), identified 128 distinct sequence types (STs), demonstrating substantial genetic diversity. This high diversity was reflected by a Shannon diversity index of 4.63 (range 0–5, where 0 indicates no diversity and 5 represents maximal diversity). Most STs were represented by only one (*n =* 94) or two isolates (*n =* 17), whereas ST56 (*n =* 11), ST2101 (*n =* 7), and ST1172 (*n =* 6) were the most frequently detected lineages. The distribution of STs was largely farm-specific, with only occasional overlap between farms (Figure 1).

The majority of isolates belonged to phylogroup B1 (74.2%), followed by phylogroups B2 (9.6%), D (6.1%), and E (5.1%). Phylogroup A was detected in four isolates, whereas phylogroups C and cryptic clade IV were each represented by two isolates. Single isolates were assigned to phylogroup G and cryptic clade III (Supplementary Table S3).

Core genome MLST (cgMLST) analysis confirmed the high genetic diversity of the isolate collection, with isolates being distributed across 22 distinct clusters (Supplementary Figure S2). Only a few small groups of closely related isolates (≤10 allelic differences) were identified, mainly within clusters 2, 7, and 22. These clusters primarily contained isolates from the same farm (e.g., farms 10, 8, and 3). In contrast, isolates from different farms generally showed larger allelic distances.

**Figure 2:**
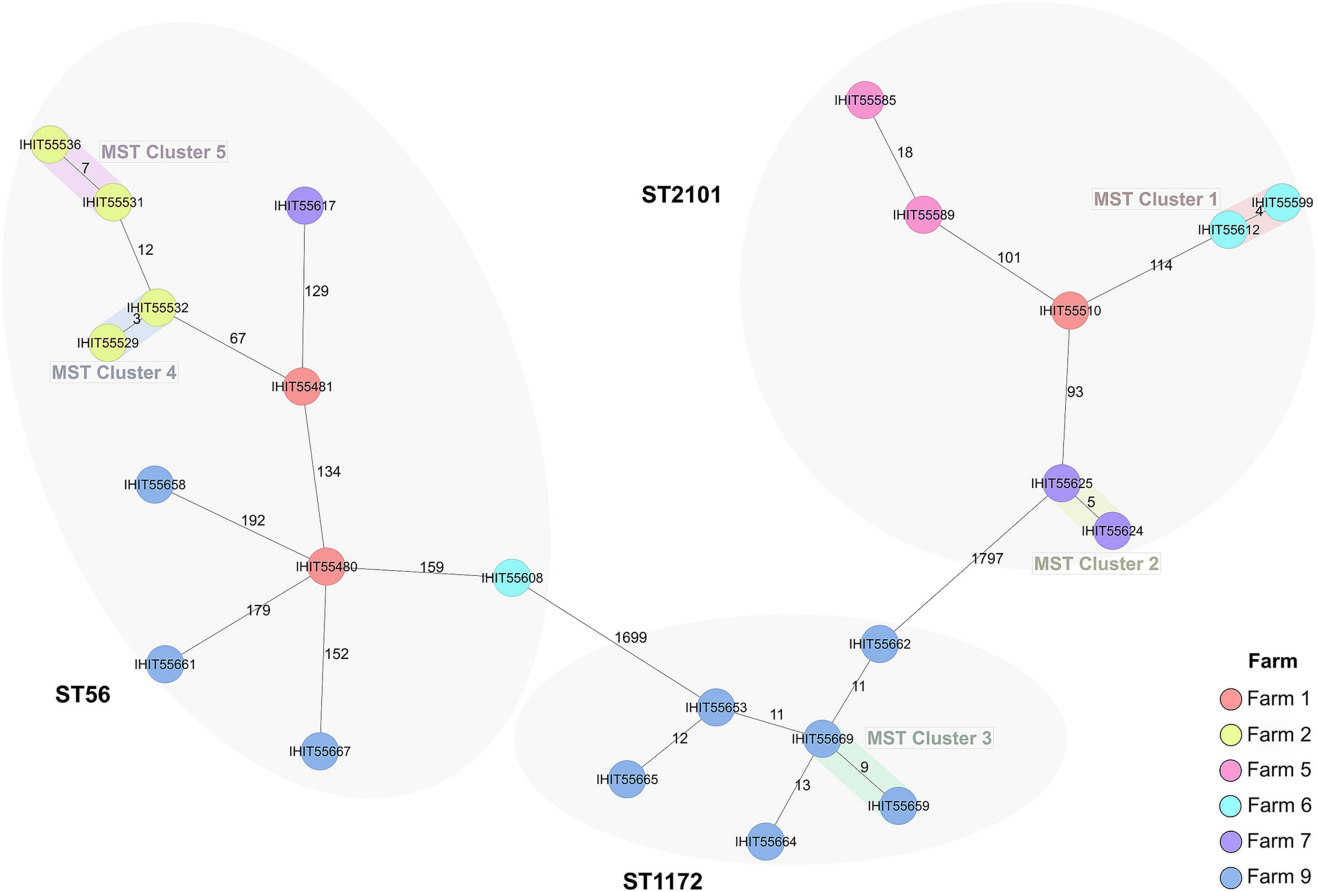
Minimum spanning tree (MST) of selected sequence types (ST56, ST1172, and ST2101), each represented by ≥5 isolates, among non-selectively obtained *E. coli* isolates from sampling round A, based on core genome multilocus sequence typing (cgMLST). Each node represents an individual isolate, and numbers on the connecting lines indicate allelic differences across the cgMLST scheme. Colors indicate the farm of origin. Shaded areas highlight isolates belonging to the same sequence type (ST).

A cgMLST-based MST of STs represented by ≥5 isolates (ST56, ST1172, and ST2101) is shown in Figure 2. It becomes evident that within-ST allelic variation is high and clusters of closely related isolates are rare and primarily confined to individual farms.

#### 3.4.2 Selectively obtained isolates

All 18 *mcr-1*-positive isolates belonged to phylogroup A and ST1684. These isolates shared an identical cgMLST complex type (CT37909) and serotype (O101:H10), indicating a clonal origin.

The 70 cefotaxime-non-WT isolates were distributed across 22 distinct STs, very often represented by only a single isolate (*n =* 12 STs). ST162 (41.4%) was predominant, followed by ST10 (8.6%), ST457 and ST1146 (each 7.1%), and ST589 (4.3%). Less frequent STs included ST155, ST224, ST1196, ST1308, ST2271 (each 2.9%), as well as ST56, ST75, ST117, ST316, ST361, ST1145, ST1246, ST1248, ST3381, ST4681, ST6951, and ST15636 (each 1.4%). The Shannon diversity index of 2.32 indicated moderate to high genetic diversity among the cefotaxime-selected isolates, although substantially lower than that observed among the non-selectively obtained isolates.

The majority of isolates belonged to phylogroup B1 (68.6%), followed by phylogroups A (11.4%), F (7.1%), and B2 and E (each 5.7%). A single isolate was assigned to phylogroup G.

### 3.5 Antimicrobial resistance (AMR) genes

Whole-genome sequencing was performed for 198 non-selectively obtained isolates [194 isolates from sampling round A and four isolates from the other sampling rounds B, C, and D, displaying non-WT phenotypes toward colistin (*n =* 1) or cefotaxime (*n =* 3)]. The SEL group comprised 15 presumptive tigecycline isolates, 18 *mcr-1*-positive isolates, 13 amikacin non-WT and 70 cefotaxime-non-WT isolates. Detailed results are provided in Supplementary Table S1.

Across both cultivation approaches, all 19 PCR-confirmed *mcr-1*-positive isolates carried the *mcr-1.1* variant located on IncI2 plasmids. Additional genes were frequently co-harbored, including those conferring resistance to tetracyclines (*tet*(A)*, n =* 19), *β*-lactams (*bla*_TEM-135_, *n =* 17), fluoroquinolones (*qnrS1*, *n =* 19), as well as GyrA mutations (S83L; *n =* 19) and fosfomycin resistance-associated GlpT mutations (E448K; *n =* 19) (Supplementary Table S1).

Among β-lactamase genes, the most common were *bla*_TEM-1_ (*n =* 47) and *bla*_TEM-135_ (*n =* 17), both class A enzymes. ESBL genes included *bla*_CTX-M-1_ (*n =* 7), *bla*_CTX-M-14_ (*n =* 2), *bla*_CTX-M-15_ (*n =* 9), *bla*_CTX-M-27_ (*n =* 1), and *bla*_CTX-M-55_ (*n =* 35). AmpC-type β-lactamases were represented by *bla*_DHA-1_ (*n =* 4) and *bla*_CMY-2_ (*n =* 4). The ubiquitous *bla_EC_* gene was detected in nearly all isolates (284/286), including the variant *bla_EC-5_* (*n =* 2), and the ampC C42T mutation was found in two isolates. Class D β-lactamase genes *bla*_OXA-1_ and *bla*_OXA-10_ were each found in one isolate (Supplementary Table S1).

Tetracycline resistance genes included *tet*(A) (*n =* 32), *tet*(B) (*n =* 3), and *tet*(M) (*n =* 34). Aminoglycoside resistance genes included *aadA1* (*n =* 39), *aadA2* (*n =* 11), *aadA5* (*n =* 1), *aph(6)-Id* (*n =* 52), *aph(3″)-Ib* (*n =* 52), *aph(3′)-Ia* (*n =* 38), *aac(3)-IId* (*n =* 35), and *sat2* (*n =* 1) (Supplementary Table S1).

Fluoroquinolone resistance was associated with the plasmid-located genes *qnrS1* (*n =* 27), *qnrB4* (*n =* 4), and *qnrB19* (*n =* 5), as well as one or more of the following chromosomal mutations in *gyrA* (S83L, *n =* 59; D87N, *n =* 39), *parC* (S80I, *n =* 39; S57T, *n =* 3), and *parE* (D475E, *n =* 7; S458A, *n =* 5; I355T, *n =* 3; L416F, *n =* 3). The *qnrB19* gene (*n =* 5) was detected on small (~1.3 kb) contigs for which no known replicon type was identified. The *qnrB4* gene was identified on IncFIA plasmids (*n =* 2) and on multireplicon IncFIA/IncFIC plasmids (*n =* 2). The *qnrS1* gene was found on IncFIB plasmids (*n =* 3) and IncY plasmids (*n =* 3); however, the majority of *qnrS1*-carrying isolates were either untypeable by plasmid replicon typing (*n =* 13) or showed chromosomal localization (*n =* 8) (Supplementary Table S1).

Other resistance determinants included genes mediating resistance to phenicols (*cmlA1, cmlA5, catA1, floR*; *n =* 42), sulfonamides (*sul1, sul2, sul3*; *n =* 60), and trimethoprim (*dfrA1, dfrA5, dfrA7, dfrA12, dfrA14, dfrA17, dfrA36, dfrA51*; *n =* 62). Less frequently detected were genes conferring resistance to rifampin (*arr-2*; *n =* 1), lincosamides (*lnu(F); n =* 25), and macrolides (*mph(A)*; *n =* 11). Fosfomycin resistance was associated with both the *fosA7.5* gene (*n =* 3) and chromosomal mutations in GlpT (E448K; *n =* 237), UhpT (E350Q; *n =* 37), and CyaA (S352T; *n =* 17) (Supplementary Table S1).

No *tet*(X) genes or acquired 16S rRNA methyltransferase genes (e.g., *armA* or *rmtB*) were detected in isolates recovered from tigecycline- or amikacin-containing media.

### 3.6 Pathotype prediction and phylogenetic grouping of *E. coli* isolates

Virulence-associated gene analysis of 286 sequenced isolates identified intestinal pathogenic *E. coli* (InPEC) on eight of 10 farms. STEC was the most frequently identified pathotype (*n =* 15), followed by AEEC (*n =* 6). One isolate (O63:H6, ST583) was classified as an AEEC/STEC hybrid strain carrying both *stx2f* and the intimin gene *eae*. STEC also encoded the *stx* subtypes *stx1c*, *stx2b*, and *stx2d*. Farms 6, 7, and 10 harbored the highest number and diversity of AEEC and STEC isolates, encompassing multiple distinct STs (Supplementary Tables S2, S3).

ExPEC-associated genes were detected in four isolates from farms 1, 2, 5, and 10. These isolates carried *iutA*, *papBCDFHIJKX*, *focACDFGI*, and *sfa*/*afa* variants (*sfaBCDEFGHXSY*, *afaABCDEVIII, afaF-VII*), as well as toxin genes (*cnf1*, *hlyABCD*, *cdtABC*). Three isolates were classified as UPEC and one as SEPEC, according to the criteria applied in this study. Farms 5 and 10 harbored both InPEC and ExPEC strains, demonstrating higher pathovar diversity. Overall, InPEC prevalence exceeded that of ExPEC (Supplementary Table S2).

MLST analyses revealed genetic diversity among STEC isolates, with ST2101 being the most common sequence type (*n =* 6; farms 5–7; phylogroup B1). AEEC isolates were primarily associated with ST589 (farm 4), ST2678 (farm 6), and ST526 and ST122 (farm 10), all belonging to phylogroup B2. The four ExPEC isolates were assigned to distinct STs (ST12, ST58, ST75, and ST141) across phylogroups B1 and B2 (Supplementary Table S2).

cgMLST analyses provided additional resolution, allowing discrimination among isolates within identical STs. For instance, ST2101 isolates were divided into five distinct complex types (CT37930, CT38016, CT38024, CT62117, CT62118), revealing within-clonal diversity. Similarly, ST56 isolates exhibited even greater diversity, comprising ten CTs and ST675 isolates were assigned into two CTs (Supplementary Table S3). Twenty-three isolates that could not be assigned to existing STs were submitted to EnteroBase,^3^ resulting in 16 novel STs (ST18320–ST18327 and ST18330–ST18337) (Supplementary Table S3).

Phylogenetic analysis indicated that phylogroup B1 predominated among InPEC isolates, particularly STEC. Most STEC isolates belonged to phylogroup B1, whereas the *stx2f*/*eae*-positive STEC isolate (O63:H6/ST583) (Bugarel et al., 2011) was assigned to phylogroup B2. All AEEC isolates were exclusively associated with phylogroup B2. The four ExPEC isolates were evenly distributed between phylogroups B1 and B2 (Supplementary Table S3).

### 3.7 MLVA analysis of non-selectively obtained isolates

MLVA was used to assess the genetic diversity of 706 non-SEL isolates representing individual animals from 10 SAC farms. Because of financial constraints, whole-genome sequencing (WGS) of non-selectively obtained isolates was mainly restricted to sampling round A, with four additional isolates from later rounds included. Therefore, MLVA provided an initial overview of the genetic diversity within the entire non-SEL isolate collection.

MLVA analysis demonstrated considerable genetic diversity and identified recurring profiles within individual farms, consistent with local strain circulation. Multiple MLVA profiles were particularly observed on farms with larger sample sizes (*n =* 73–80) (farms 1–4, 6–8, and 10) (Supplementary Figures S1A–1D, S1F–1H, S1J). For example, SACs 2.15 and 8.18 harbored three distinct profiles across different sampling rounds (A–D) (Supplementary Figures S1B, S1H).

Despite this diversity, identical banding profiles across all VNTR loci (i.e., identical MLVA profiles) were detected within individual farms, suggesting local clonal expansion or persistence. For example, SACs from farm 1 (SACs 1.1, 1.21, 1.26, 1.29, and 1.30) and farm 8 (SACs 8.12, 8.16, 8.20, 8.22, and 8.23) shared identical profiles across multiple sampling rounds (A–B) and A–C, respectively, consistent with within-farm transmission (Supplementary Figures S1A, S1H).

In contrast, farms with smaller sample sizes (*n =* 20–69) (farms 5 and 9) showed high strain diversity without dominant profiles (Supplementary Figures S1E, S1I).

On farm 10, repeated sampling across four sampling rounds revealed both profile stability and temporal turnover, with some animals maintaining consistent MLVA profiles across sampling rounds (e.g., SAC 10.2), while others (e.g., SACs 10.16 and 10.17) exhibited shifts to unrelated profiles (Supplementary Figure S1J).

Profile distribution varied between farms. Farms 1 and 8 showed dominance of recurring MLVA profiles. For example, identical MLVA profiles were detected across sampling rounds A–C in multiple SACs (e.g., SAC 1.1; SACs 1.21, 1.26, 1.29, and 1.30; and SACs 1.44, 1.45, and 1.50) (Supplementary Figure S1A). In contrast, farms 5, 7, and 9 exhibited broader strain diversity without dominant profiles (Supplementary Figures S1E, S1G, and S1I). These findings illustrate differences in the population structure of *E. coli* between farms, with both recurring clonal profiles and diverse strain populations observed.

### 3.8 Comparison of MLVA and whole-genome sequencing

A comparison of MLVA banding profiles with WGS-derived data for the 194 non-SEL *E. coli* isolates from the initial sampling round A highlighted both concordance and discrepancies between the two methods. MLVA differentiated the isolates into 30 unique banding profiles (100% threshold), whereas WGS resolved 126 multilocus STs and 164 unique cgMLST complex types (CTs), demonstrating a markedly higher resolution of genetic diversity (Supplementary Figure S1K). Consistent with the differences observed, the extensive ST diversity and high allelic variation revealed by WGS analyses (Figure 1 and Supplementary Figure S2) underscore the substantially higher resolution of WGS compared to MLVA.

In several instances, MLVA clustering was concordant with WGS results, supporting clonal relatedness. For example, farm 10 isolates 10A01, 10A02, and 10A04 shared identical MLVA profiles and were confirmed as clonal by WGS (ST1248, CT5377, Ont:H21, phylogroup B1). Similarly, farm 10 isolates 10A10, 10A14, 10A19, and 10A20 clustered by MLVA and were also indistinguishable by WGS (ST2164, CT66919, O74:H28, phylogroup B1). Concordant MLVA-WGS clustering was further observed in farm 8 isolates 8A07 and 8A09, which shared identical MLVA profiles and belonged to ST1727 (CT66909, Ont:H7, phylogroup B1), as well as in farm 3 isolates 3A04 and 3A06 (ST1704, CT37963, Ont:H7, phylogroup B1) and farm 4 isolates 4A02 and 4A03 (ST1726, CT37973, Ont:H31, phylogroup B1).

However, MLVA sometimes failed to discriminate genetically distinct strains. For example, farm 2 isolates 2A15 and 2A16 shared identical MLVA profiles and the same sequence type ST56 but were assigned to distinct CTs (CT37960 and CT37957). Similarly, farm 9 isolates 9A03, 9A09, and 9A19 shared identical MLVA profiles, ST1172 and Ont:H48 but differed in CTs (CT37896, CT37902).

Conversely, some isolates displayed distinct MLVA profiles despite close genomic relatedness, including isolates 2A13 and 2A16 (ST56, CT37957, Ont:H21, phylogroup B1), and isolates 6A01 and 6A05 (ST154, CT37935, Ont:H25, phylogroup B1).

When comparing isolates across farms (Supplementary Figure S1K), MLVA frequently grouped strains from different farms into identical profiles (100% identity), suggesting potential inter-farm transmission. However, sequence-based analyses demonstrated that many apparent cross-farm similarities were misleading, as isolates often differed in ST, serotype, or phylogroup despite identical MLVA patterns. For example, isolates 4A15 (farm 4) and 10A15 (farm 10) were indistinguishable by MLVA but differed by WGS (ST40, CT38003, Ont:H21, phylogroup B1 vs. ST58, CT66923, Ont:H4, phylogroup B1). Similarly, isolates 1A10 (farm 1) and 3A18 (farm 3) shared MLVA profiles but were distinct by WGS (ST25, CT37921, Ont:H2, phylogroup B1 vs. ST2, CT37969, Ont:H18, phylogroup D). Additional discrepancies between MLVA clustering and WGS-based typing were observed across multiple farms in Supplementary Figure S1K.

## 4 Discussion

This study provides a comprehensive genomic characterization of *Escherichia coli* from South American camelids (SACs) in Germany and demonstrates that these animals harbor genetically diverse *E. coli* populations, including isolates carrying clinically relevant antimicrobial resistance determinants and zoonotic virulence traits.

Overall, antimicrobial susceptibility testing based on epidemiological cutoff values (ECOFFs) revealed a low prevalence of non-WT phenotypes among non-selectively obtained isolates. This applies to a panel of 14 antimicrobial agents relevant to veterinary and human medicine, including compounds used in SAC husbandry, although their specific importance varies between sectors (Giguère et al., 2013; WHO, 2019; Commission Implementing Decision, 2020; BVL, 2023). The highest proportions of non-WT isolates were observed for sulfamethoxazole, trimethoprim, tetracycline, and ampicillin, whereas non-WT phenotypes toward critically important antimicrobials, such as third-generation cephalosporins and colistin, remained rare. These findings are largely consistent with current EU surveillance data from livestock (EFSA, 2025), and suggest limited antimicrobial selection pressure in SAC populations in Germany.

However, this overall low prevalence contrasts with previous findings in German SACs using selective cultivation (González-Santamarina et al., 2022a). In line with this, the proportion of non-WT isolates in German SACs appears considerably lower than reported in South American studies (Luna et al., 2012; Barrios-Arpi et al., 2016; Carhuapoma De Cruz et al., 2020), where *E. coli* isolates frequently exhibited resistance to enrofloxacin (12%), trimethoprim/sulfonamides (6%), and gentamicin (5%). These differences likely reflect variations in production systems and animal management between regions, as SACs in South America are primarily raised for food production (Pérez et al., 2000), whereas in Germany, they are more commonly kept as hobby animals (Wagner et al., 2021). In addition, antimicrobial use in SACs in Germany is subject to strict regulatory constraints (Wagner et al., 2021). Furthermore, animals are predominantly bred locally due to EU animal health regulations (European Union, 2016). Consequently, the observed susceptibility patterns are likely shaped largely by regional factors (Neubert et al., 2021).

The proportion of non-WT phenotypes for antimicrobials commonly used in food-producing animals, such as ampicillin, sulfamethoxazole, trimethoprim, and tetracycline, ranged from 1.4 to 4.5%, which is substantially lower than reported for many livestock species in the EU (EFSA, 2025). Likewise, non-WT phenotypes toward WHO-classified “Watch” (e.g., third-generation cephalosporins) and “Reserve” (e.g., colistin) antimicrobials remained rare (0.1–0.4%), consistent with EU surveillance data (0–0.8% in livestock) (WHO, 2019; EFSA, 2025), although variation between species and countries exists, particularly for colistin (EFSA, 2025).

Despite the generally low prevalence of non-WT phenotypes in non-selectively obtained isolates, 11 isolates exhibited multidrug non-WT phenotypes (three to ten agents); however, only four isolates with cefotaxime and colistin non-WT phenotypes carried acquired resistance genes, including the ESBL gene *bla*_CTX-M-55_ and the plasmid-mediated colistin-resistance gene *mcr-1.1*. The occurrence of such determinants in livestock-associated *E. coli* has been widely reported, although many studies rely on selective cultivation (González-Santamarina et al., 2022b; Ghazawi et al., 2024; EFSA, 2025; Pavesi et al., 2025). A recent study in SACs using selective cultivation reported a low prevalence of ESBL-producing *E. coli* (~3.2%) (Pavesi et al., 2025), supporting the generally low resistance levels. In contrast, the detection in non-selectively obtained isolates in this study indicates that such determinants may persist at low frequency within the commensal population. The detection of *mcr-1.1* is of particular concern and represents, to our knowledge, the first report of this gene in SACs. A recent study identified *mcr-1*-positive *E. coli* in Old World camelids using selective enrichment (Ghazawi et al., 2024), suggesting that such approaches enhance detection of rare resistant subpopulations.

Selective cultivation further increased the detection of isolates carrying resistance determinants of public health concern (WHO, 2019). While no carbapenem non-WT *E. coli* isolates were recovered, *mcr-1*-positive isolates and cefotaxime non-WT isolates were detected. The *mcr-1*-positive isolates were largely confined to a single farm and predominantly detected during initial sampling, suggesting localized and potentially transient circulation. In contrast, cefotaxime non-WT isolates were more widely distributed, particularly on farm 7 (51%, *n =* 36), where they were detected across all sampling time points (A: 14, B: 4, C: 7, and D: 11). This pattern is consistent with previous reports on ESBL-producing *E. coli* in livestock (Dahms et al., 2015; González-Santamarina et al., 2022a) and indicates within-farm circulation.

Whole-genome sequencing of isolates from tigecycline- and amikacin-selective media did not reveal known high-level resistance determinants such as *tet*(X) genes (Sun et al., 2019) or acquired 16S rRNA methylases associated with aminoglycoside resistance (e.g., *armA*, *rmtA-H,* and *npmA*) (Caméléna et al., 2020). In addition, *tet* genes previously shown to acquire mutations associated with tigecycline resistance, including *tet*(A), *tet*(B), *tet*(K), and *tet*(M) (Tuckman et al., 2000; Linkevicius et al., 2016; Zhang et al., 2023), were not detected. Likewise, no aminoglycoside-modifying enzyme genes associated with reduced amikacin susceptibility, such as *aac(6′)-Ib*, were identified (Haldorsen et al., 2014). Although non-WT phenotypes to amikacin were phenotypically confirmed in 13 of 55 isolates, no corresponding acquired resistance genes were detected. These isolates exhibited inhibition zones close to ECOFF values, suggesting borderline phenotypes. Together with the presence of intrinsic efflux-associated genes (e.g., *acrF, emrD, emrE,* and *mdtM*), this suggests that the observed reduced susceptibility may reflect the cumulative effect of non-specific intrinsic resistance mechanisms within the isolate background rather than the acquisition of specific resistance mechanisms (Yu et al., 2020; Nasrollahian et al., 2024).

Overall, these findings highlight the strong influence of methodological approaches: non-SEL cultivation reflects background susceptibility, whereas selective methods enhance detection of rare but clinically relevant resistance phenotypes. The combination of both approaches therefore represents a robust strategy for AMR surveillance. In all *mcr-1.1*-positive isolates, the *mcr-1.1* gene was located on an IncI2 plasmid, confirming the key role of this plasmid family in global dissemination (Loayza-Villa et al., 2020; Zakaria et al., 2021). These plasmids were nearly identical to the previously described plasmid p974-MCR (GenBank accession MG825369) from food-derived *E. coli* in China, indicating widespread circulation of a conserved backbone across hosts and regions.

The observed prevalence detected by colistin-selective screening (2.5%; 18/706) is comparable to veal calves but lower than in pigs and poultry (Irrgang et al., 2016; Göpel et al., 2024a). The localized and transient detection of *mcr-1*-positive isolates suggests short-term colonization rather than stable establishment. Possible explanations include introduction via environmental sources (e.g., contaminated feed, livestock contact, and human-mediated vectors), clonal expansion, or horizontal transfer followed by plasmid loss in the absence of selection pressure (Randall et al., 2018; Viñes et al., 2021; Lin et al., 2022). Similar dynamics have been reported in livestock systems (Randall et al., 2018). Given that colistin is classified as a WHO “Reserve” antimicrobial, the detection of transferable *mcr-1* underscores the relevance of SACs within a One Health surveillance framework.

In addition to antimicrobial resistance, SACs carried several intestinal pathogenic *E. coli* pathotypes, particularly STEC and AEEC. The predominance of STEC among clinically healthy animals indicates asymptomatic intestinal carriage and confirms previous findings (González-Santamarina et al., 2022a). SACs may therefore act as silent reservoirs for STEC, similar to other species (Mori et al., 2014; Persad and LeJeune, 2014; Rimac Beltrán, 2015; Maturrano et al., 2018; Siuce et al., 2020). The detection of serotype O11:H25 suggests circulation of non-classical STEC serotypes beyond major priority groups (European Centre for Disease Prevention and Control, 2012; Pătrînjan et al., 2024). The presence of *stx2d* further highlights a potential zoonotic risk of SAC-associated STEC. While *stx1c*, *stx2b*, and *stx2f* are typically associated with mild or asymptomatic human infections, *stx2d* is linked to severe human disease such as hemorrhagic colitis and hemolytic uremic syndrome (HUS) (Jajarmi et al., 2018).

Genomic analyses revealed high diversity among STEC isolates, including microevolution within ST2101. Of particular relevance was the detection of an *stx2f*/*eae*-positive O63:H6/ST583 isolate, a lineage previously associated with human infections (Den Ouden et al., 2023). Although *stx2f*-positive STEC, originally detected in pigeons, were historically considered to possess lower pathogenic potential, increasing evidence links these strains to human disease, including severe clinical outcomes such as HUS (Ohmura et al., 1993; Schmidt et al., 2000; Gill et al., 2022; Den Ouden et al., 2023). Comparative studies have shown that pigeon-derived isolates differ genetically from human-associated strains, suggesting that pigeons may not represent a primary source of human infection (van Hoek et al., 2019). Moreover, human *stx2f*-positive isolates are predominantly associated with serotypes/STs O63:H6/ST583, O113:H6/ST121, or O125:H6/ST583, whereas pigeon isolates are mainly O4:H2/ST20 or O45:H2/ST20 (van Hoek et al., 2019). The detection of a strain sharing the same serotype and sequence type associated with human infections suggests that llamas and alpacas may represent alternative reservoirs for *stx2f*-positive STEC. The predominance of phylogroup B1 among STEC isolates from SACs observed in this study is consistent with previous reports showing that this pathotype is commonly associated with phylogroup B1 across diverse host species, including cattle, small ruminants, and wildlife (Ndegwa et al., 2022; Tomeh et al., 2023).

Atypical EPEC (aEPEC), which are characterized by the presence of *eae* and absence of *bfp* and represent a subgroup of AEEC (attaching and effacing *E. coli*), were identified exclusively in alpacas. As mentioned before, all isolates originated from clinically healthy animals, supporting the concept of asymptomatic carriage in SACs. Similar observations have been reported in studies from South America, where various intestinal pathogenic *E. coli* pathotypes, including EPEC (both atypical and typical), and STEC, have been identified in both healthy and diarrheic alpacas (Mori et al., 2014; Rimac Beltrán, 2015; Maturrano et al., 2018; Siuce et al., 2020). All AEEC isolates were assigned to phylogroup B2. This finding contrasts with studies in other host species, where AEEC strains were distributed across multiple phylogroups, including A, B1, B2, D, and E (Fröhlicher et al., 2008; Hernandes et al., 2020; Piryae et al., 2023). The exclusive association with B2 in SACs may therefore represent a host- or region-specific feature rather than a universal phylogenetic tendency of the pathotype.

In addition, four ExPEC-associated isolates were detected, further expanding the zoonotic risk profile. Although these isolates carried virulence gene combinations associated with UPEC and SEPEC pathotypes, their potential contribution to clinical infections remains difficult to assess. This is because no universally accepted single gene signature exists for ExPEC pathotypes (including UPEC, SEPEC, or APEC), in contrast to well-defined markers such as *stx* for STEC or *eae/bfp* for EPEC. The isolates identified in this study may nonetheless be considered potentially pathogenic, as they carried multiple virulence-associated genes linked to extraintestinal pathogenicity, such as *iutA*, *papBCDFHIJKX*, *focACDFGI*, and *sfa*/*afa* variants (*sfaBCDEFGHXSY*, *afaABCDEVIII, afaF-VII*), as well as toxin genes (*cnf1*, *hlyABCD*, *cdtABC*). Their presence in the gut microbiota of SACs suggests potential reservoirs for ExPEC lineages with zoonotic relevance.

MLVA revealed both clonal groups and high genetic diversity among *E. coli* isolates from SACs, reflecting complex population structures. Whole-genome sequencing further demonstrated substantial sequence type (ST) diversity, with most STs represented by only a single isolate, indicating a highly heterogeneous *E. coli* population in SACs. The predominance of ST56, ST1172, and ST2101 within individual farms suggests localized circulation of specific lineages rather than widespread inter-farm dissemination. Within farms, identical or closely related profiles suggest local transmission or environmental persistence (Murphy et al., 2008), whereas diverse profiles within individual animals indicate repeated exposure to heterogeneous *E. coli* strains (Khare et al., 2020). Differences in strain diversity between farms likely reflect variations in management practices, environmental contamination, and animal contact patterns. These findings suggest that *E. coli* population structure in SACs is shaped by both within-farm transmission and farm-specific factors. Comparison with whole-genome sequencing (WGS) showed that MLVA may overestimate genetic relatedness, particularly between farms. While MLVA is useful for rapid screening, it should be complemented by WGS for accurate epidemiological inference (Caméléna et al., 2019). However, WGS remains constrained by cost, time requirements, and bioinformatics capacity (Caméléna et al., 2019).

Several limitations should be considered. First, only a subset of isolates underwent whole-genome sequencing. Genomic characterization of the non-selectively obtained *E. coli* population was primarily based on isolates from the initial sampling round, whereas selectively obtained resistant isolates originated from all sampling rounds. Second, only one colony per sample was analyzed in the non-SEL approach, potentially underestimating within-host diversity. Third, environmental samples and human contact samples were not included, limiting direct conclusions regarding transmission pathways within the One Health interface. Fourth, repeated sampling of individual animals may have influenced prevalence estimates, and the relatively small number of farms and unequal numbers of samples between farms and sampling rounds may limit the generalizability of the findings. Finally, only clinically healthy animals were included, and differences in farm management practices and co-housed animal species were not assessed.

Nevertheless, the present study demonstrates that SACs can harbor zoonotically relevant and antimicrobial-resistant *E. coli* lineages despite low overall resistance prevalence. Given the increasing popularity of SACs and their close contact with humans in recreational settings, these animals should be considered in future One Health surveillance strategies.

## 5 Conclusion

This study demonstrates that SACs in Germany harbor highly diverse *E. coli* populations with overall low background antimicrobial resistance levels but sporadic occurrence of clinically relevant resistance determinants and zoonotic pathotypes. The detection of ESBL-producing isolates, transferable *mcr-1.1*-mediated colistin resistance, STEC-associated lineages, and ExPEC-like isolates highlights the potential role of SACs as currently underrecognized reservoirs within the One Health interface.

The combined use of selective and non-SEL cultivation together with whole-genome sequencing provided complementary insight into both the background *E. coli* population and low-frequency resistant subpopulations. These findings support the inclusion of SACs in future antimicrobial resistance and zoonotic pathogen surveillance programs, particularly in settings involving close human–animal contact.

## Data Availability

Data are contained in the article and supplementary material. The datasets presented in this study can be found in online repositories. The genomic sequence data are deposited in the European Nucleotide Archive (ENA) online repository under study accession number PRJEB115497: https://www.ebi.ac.uk/ena.

## Footnotes

1 version 2.0, http://rast.nmpdr.org

2 http://clermontyping.iame-research.center/index.php, accessed 03 July 2025

3 https://enterobase.warwick.ac.uk/, accessed 30 November 2025
