# Supplementary material for: Antimicrobial susceptibility and virulence traits of Escherichia coli in healthy South American camelids in Germany
Source: Front Microbiol. 2026 Jul 21;17:1898372. doi: 10.3389/fmicb.2026.1898372 (PMC13433843; doi:10.3389/fmicb.2026.1898372)
Supplement: Supplementary file 1 [file Data_Sheet_1.PDF]

Supplementary Material

1 Supplementary Figures and Tables

1.1 Supplementary Figures

Supplementary Figure 1. MLVA patterns of 194 *E. coli* isolates recovered on non-selective medium from fecal samples of South American camelids during sampling rounds A–D. (A–J) Isolates from farms 1–10. (K) Overview dendrogram including all 194 *E. coli* isolates obtained from sampling round A across all farms. Strain information (farm number, sampling round, sample number, animal code, phylogroup, sequence type (ST), clonal complex (CC), core-genome multilocus sequence typing complex type (cgMLST CT), and serotype) is indicated on the right. Green dotted lines indicate the 75% similarity cutoff used to define clusters (Ranjbar et al., 2017). Clusters were only designated when they contained at least two isolates.

Supplementary Figure 2. Minimum spanning tree (MST) of 194 non-selectively obtained *E. coli* isolates from sampling round A, based on core-genome multilocus sequence typing (cgMLST) allelic distances. Each node represents an individual isolate, and number on the connecting lines indicate allelic differences across the cgMLST scheme. Colors indicate the farm of origin.

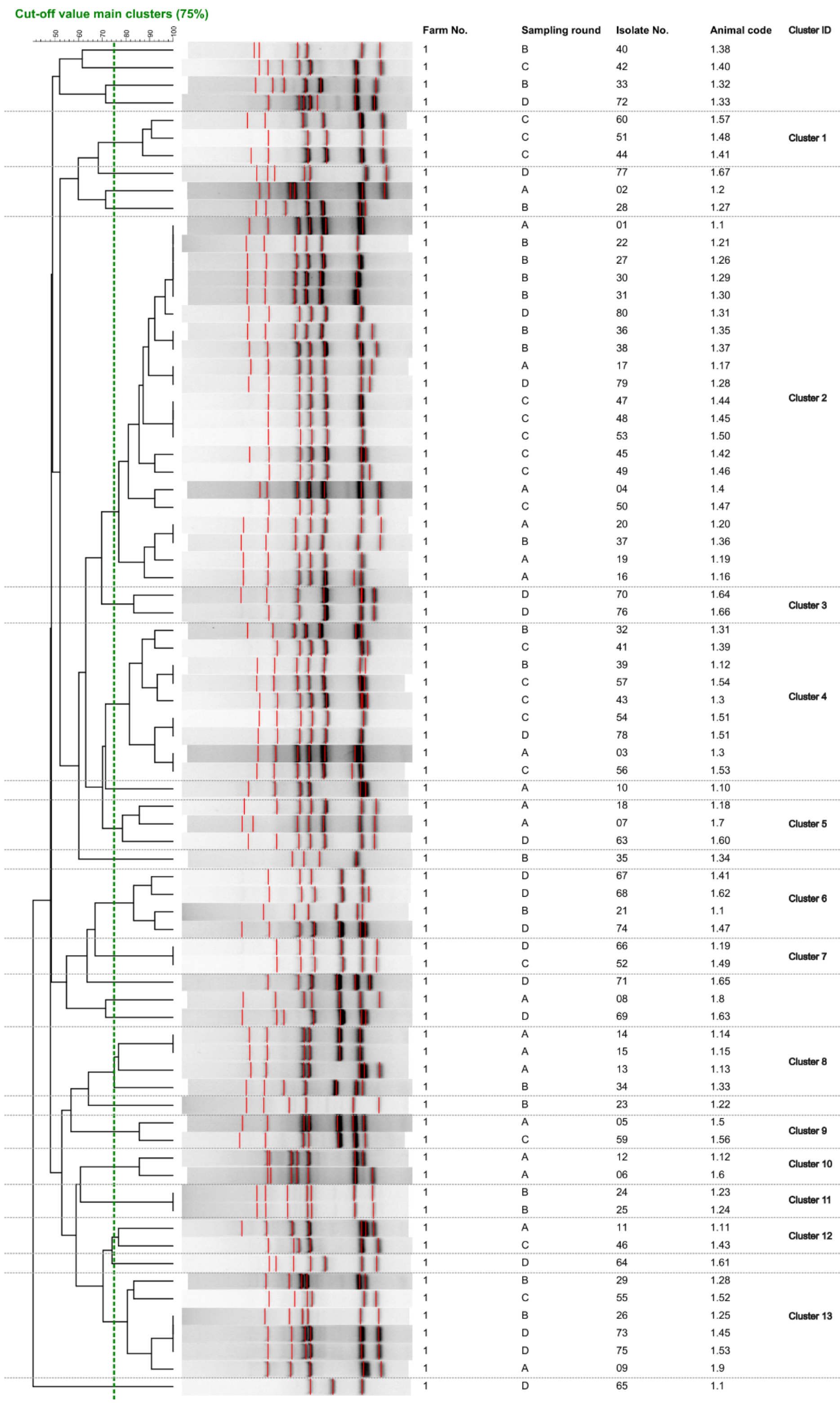

Supplementary Figure 1A. MLVA patterns of 77 *E. coli* isolates recovered on non-selective medium from fecal samples of South American camelids on farm 1 during sampling rounds A–D. Green dotted lines indicate the 75% similarity cutoff used to define clusters (Ranjbar et al., 2017). Clusters were only designated when they contained at least two isolates.

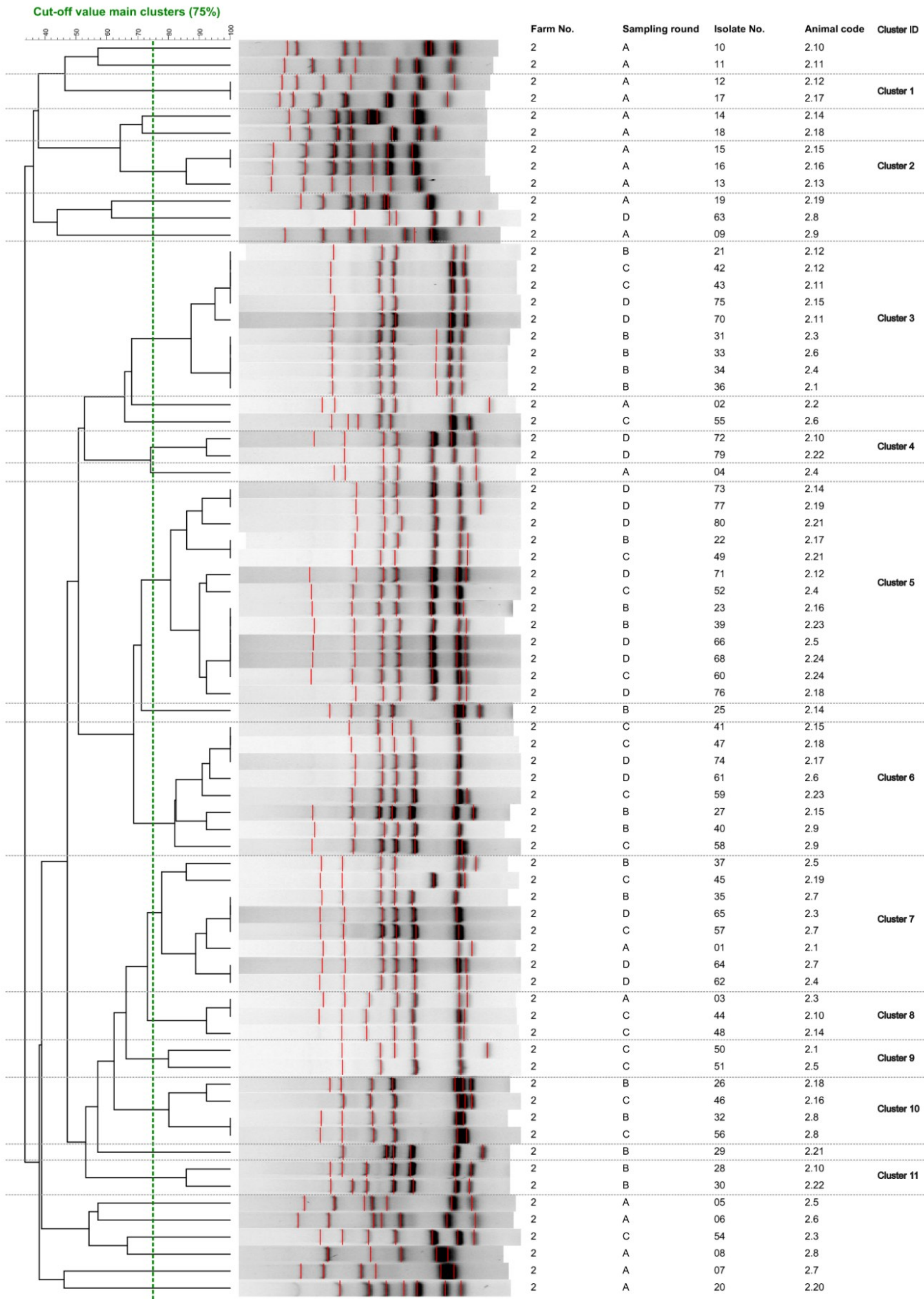

Supplementary Figure 1B. MLVA patterns of 74 *E. coli* isolates recovered on non-selective medium from fecal samples of South American camelids on farm 2 during sampling rounds A–D. Green dotted lines indicate the 75% similarity cutoff used to define clusters (Ranjbar et al., 2017). Clusters were only designated when they contained at least two isolates.

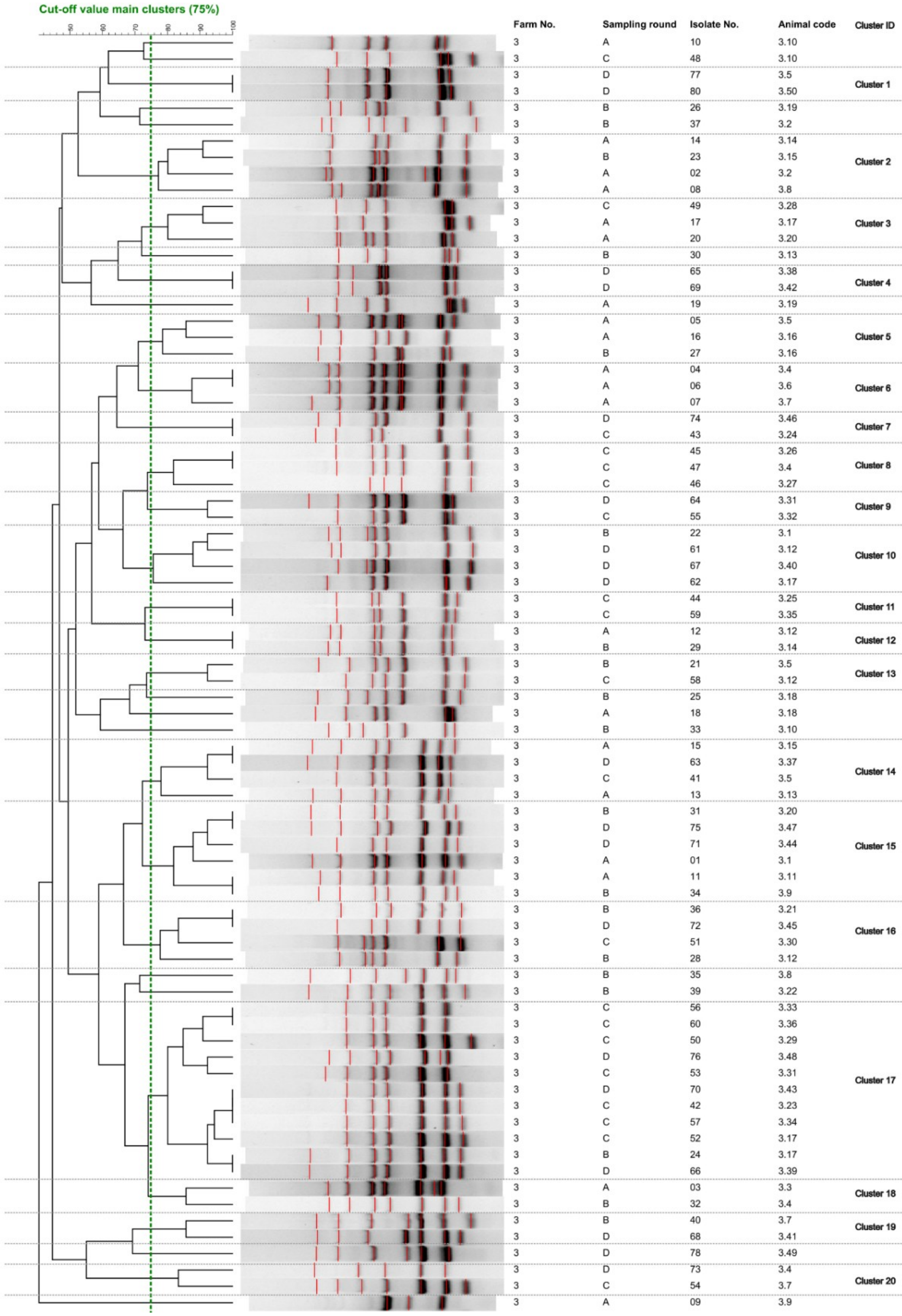

Supplementary Figure 1C. MLVA patterns of 78 *E. coli* isolates recovered on non-selective medium from fecal samples of South American camelids on farm 3 during sampling rounds A–D. Green dotted lines indicate the 75% similarity cutoff used to define clusters (Ranjbar et al., 2017). Clusters were only designated when they contained at least two isolates.

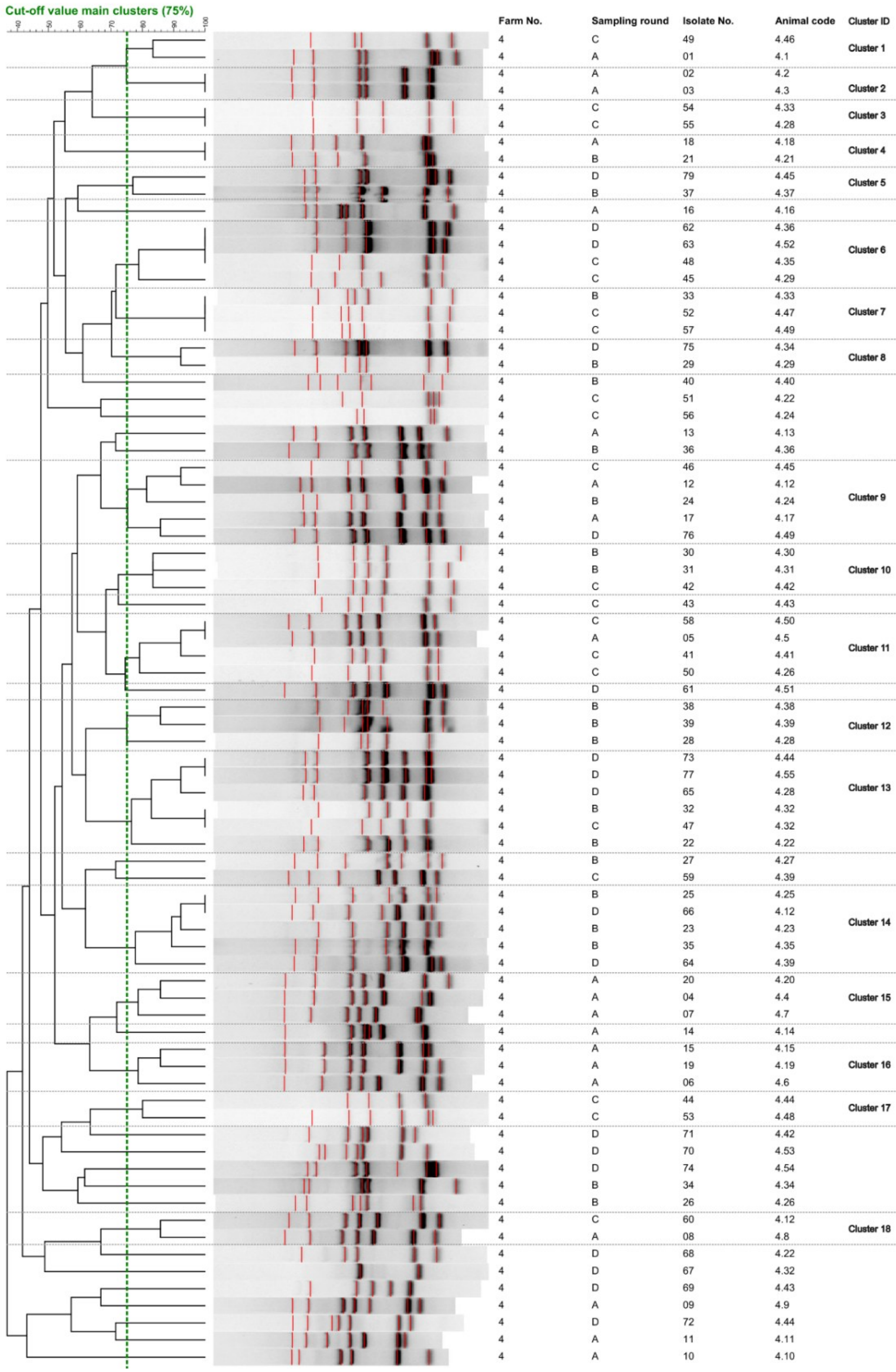

Supplementary Figure 1D. MLVA patterns of 78 *E. coli* isolates recovered on non-selective medium from fecal samples of South American camelids on farm 4 during sampling round**s** A–D. Green dotted lines indicate the 75% similarity cutoff used to define clusters (Ranjbar et al., 2017). Clusters were only designated when they contained at least two isolates.

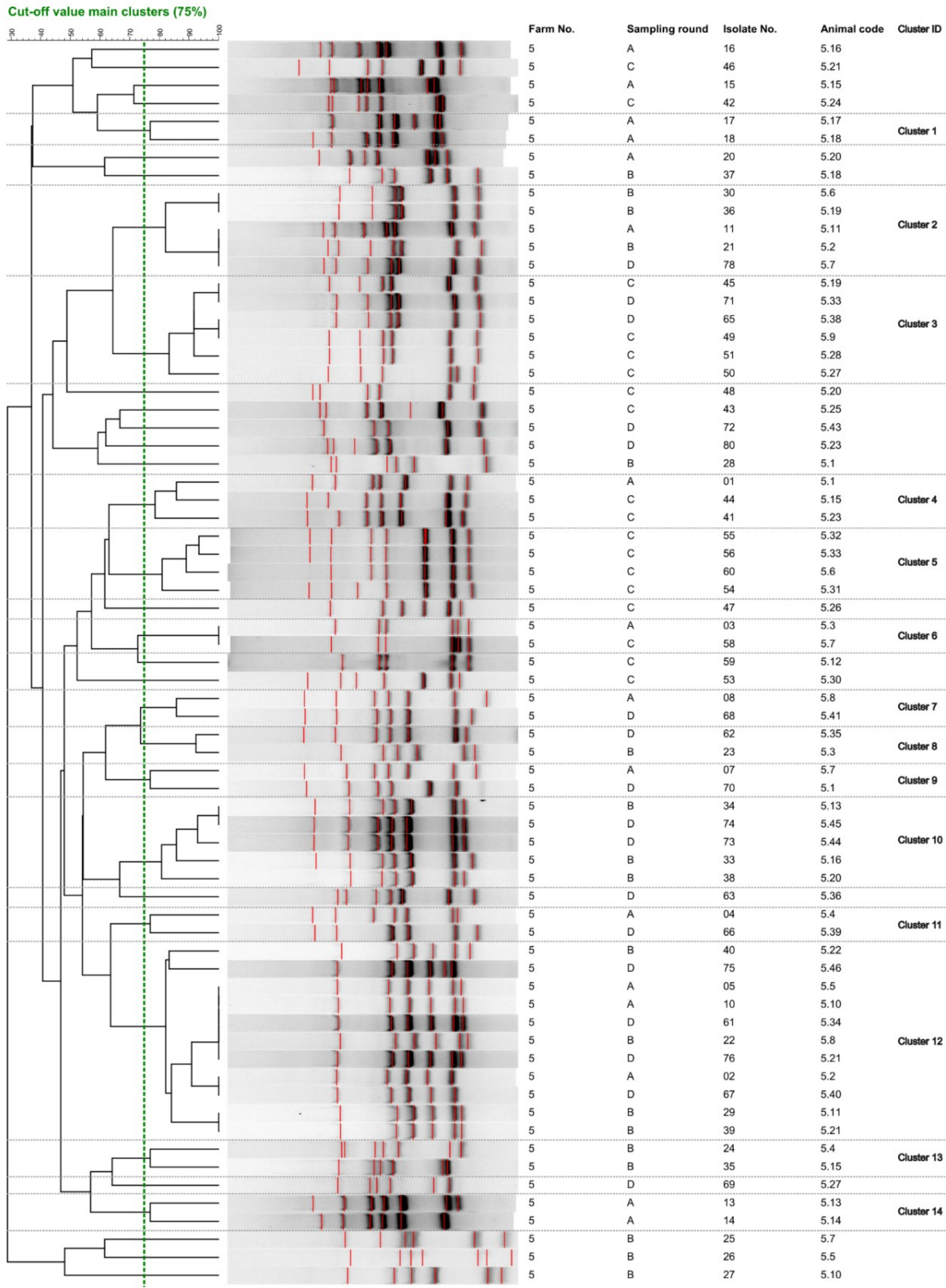

Supplementary Figure 1E. MLVA patterns of 69 *E. coli* isolates recovered on non-selective medium from fecal samples of South American camelids on farm 5 during sampling rounds A–D. Green dotted lines indicate the 75% similarity cutoff used to define clusters (Ranjbar et al., 2017). Clusters were only designated when they contained at least two isolates.

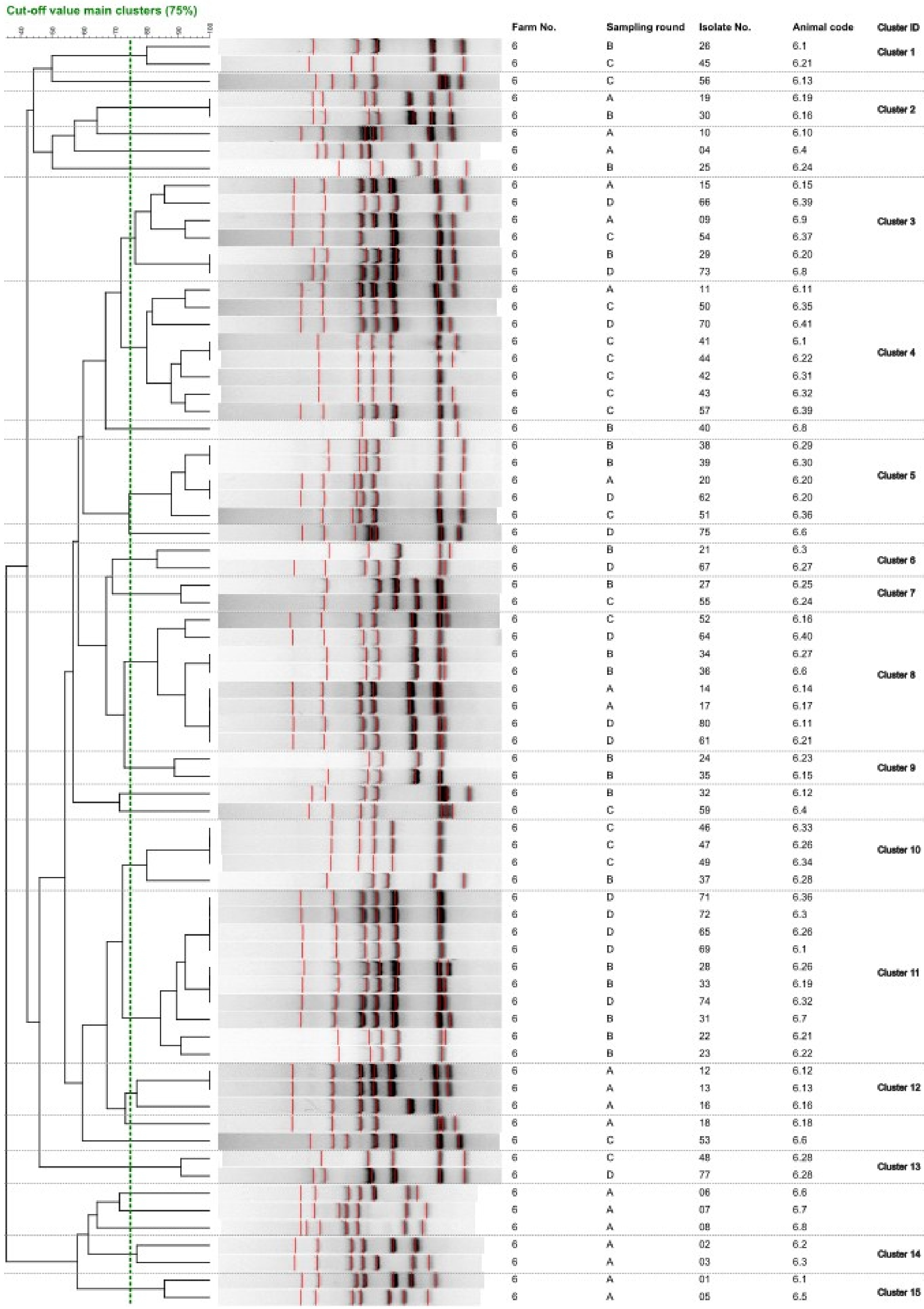

Supplementary Figure 1F. MLVA patterns of 73 *E. coli* isolates recovered on non-selective medium from fecal samples of South American camelids on farm 6 during sampling rounds A–D. Green dotted lines indicate the 75% similarity cutoff used to define clusters (Ranjbar et al., 2017). Clusters were only designated when they contained at least two isolates.

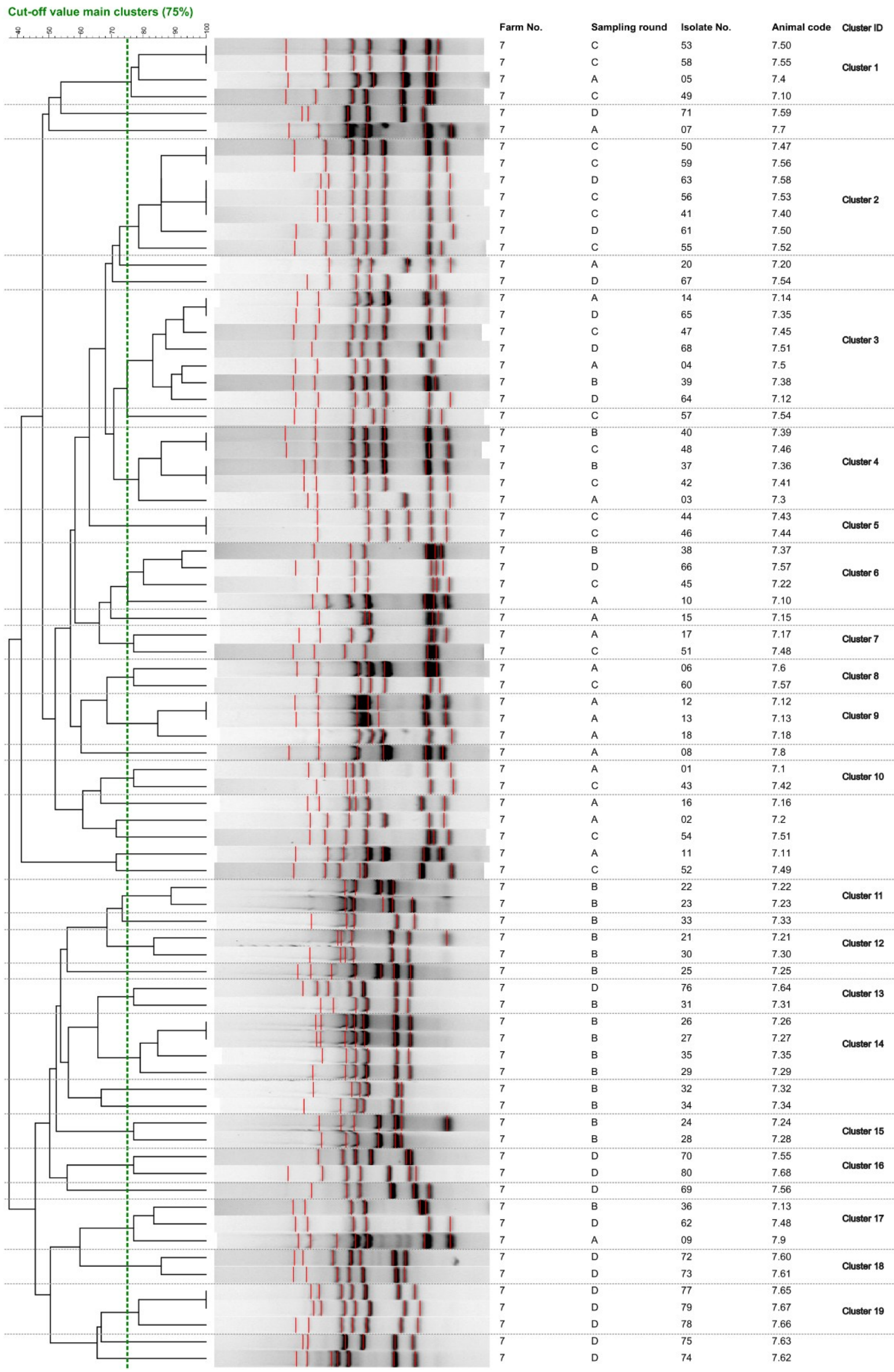

Supplementary Figure 1G. MLVA patterns of 79 *E. coli* isolates recovered on non-selective medium from fecal samples of South American camelids on farm 7 during sampling rounds A–D. Green dotted lines indicate the 75% similarity cutoff used to define clusters (Ranjbar et al., 2017). Clusters were only designated when they contained at least two isolates.

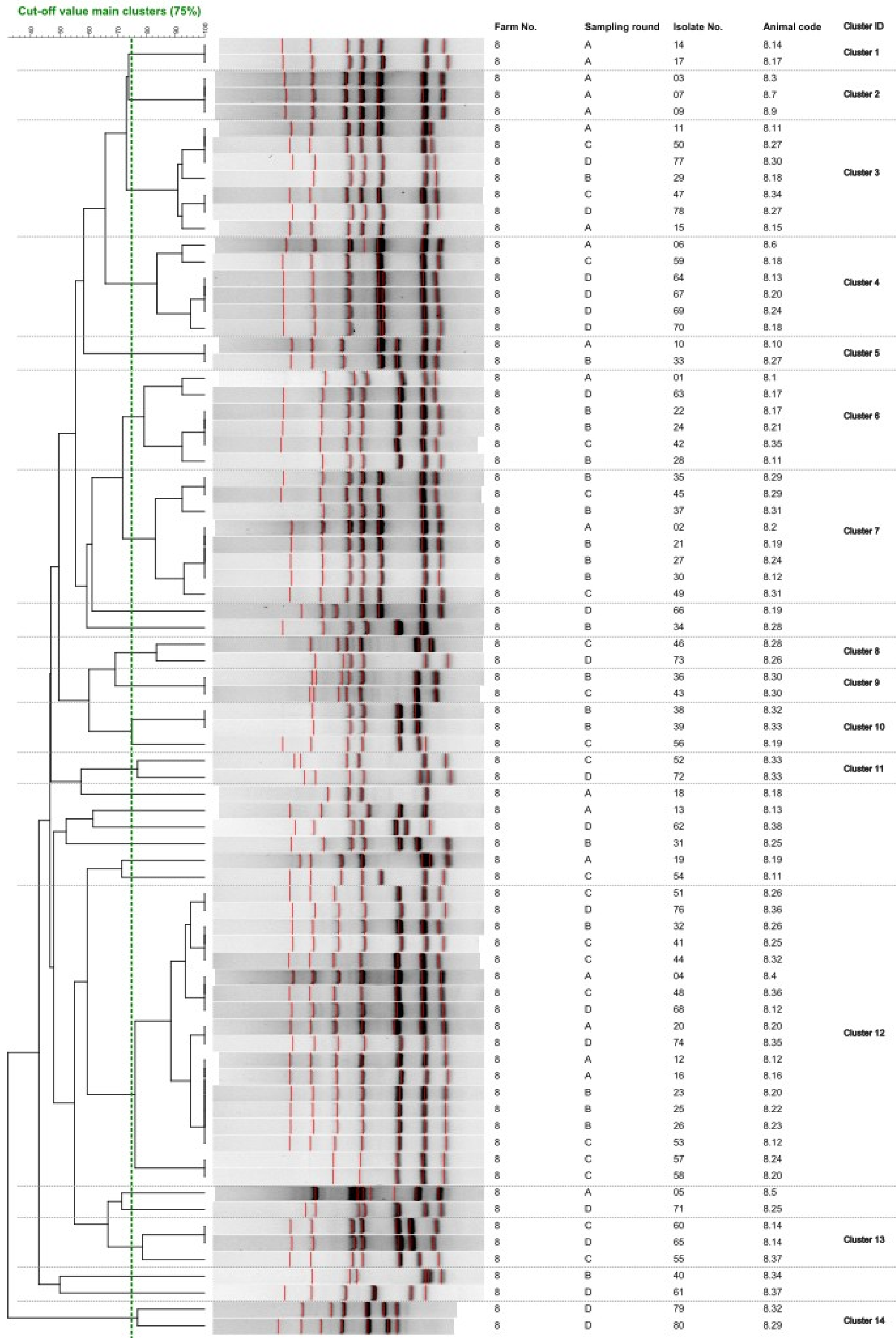

Supplementary Figure 1H. MLVA patterns of 78 *E. coli* isolates recovered on non-selective medium from fecal samples of South American camelids on farm 8 during sampling rounds A–D. Green dotted lines indicate the 75% similarity cutoff used to define clusters (Ranjbar et al., 2017). Clusters were only designated when they contained at least two isolates

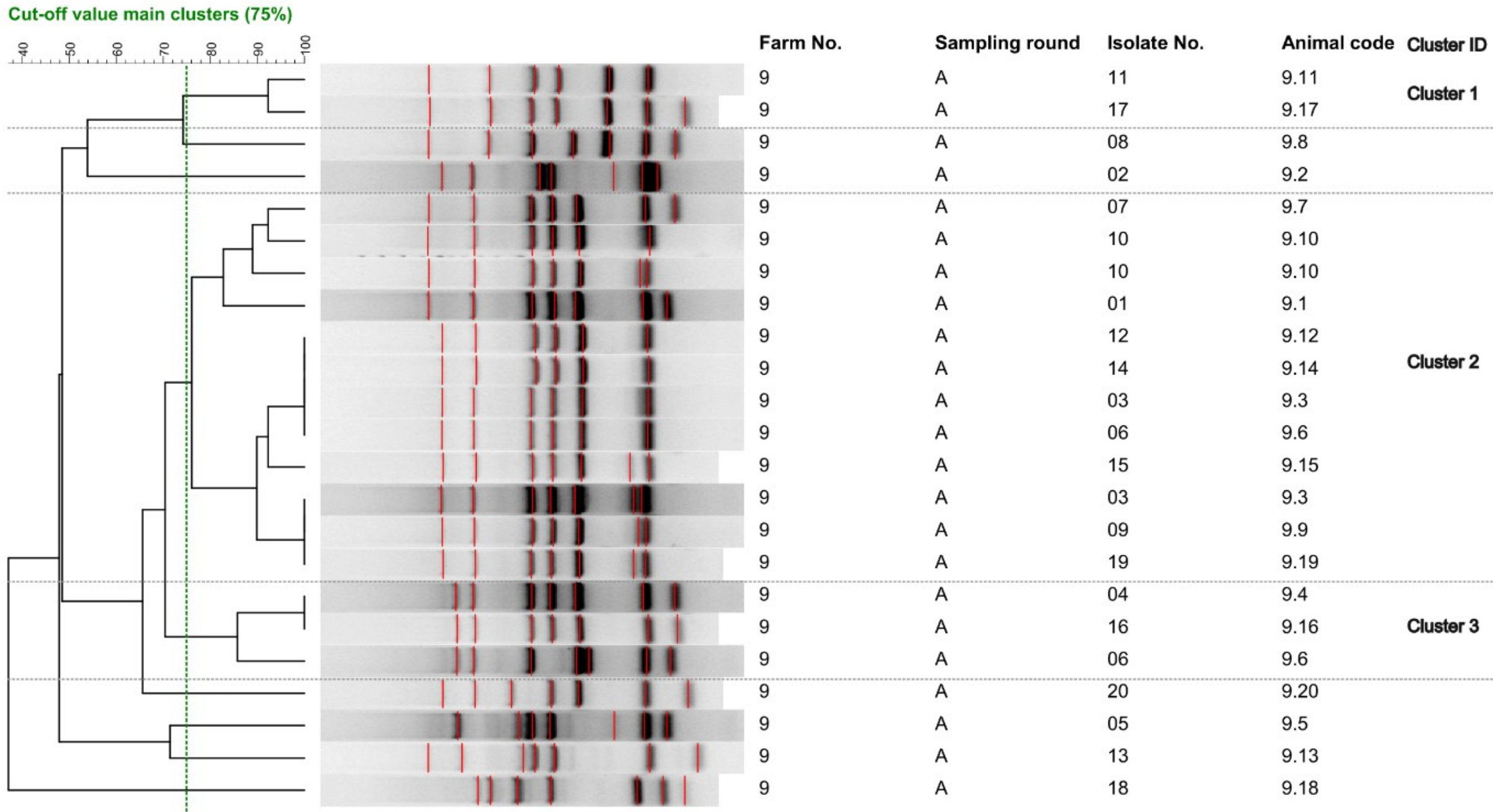

.

Supplementary Figure 1I. MLVA patterns of 20 *E. coli* isolates recovered on non-selective medium from fecal samples of South American camelids on farm 9 during sampling rounds A–D. Green dotted lines indicate the 75% similarity cutoff used to define clusters (Ranjbar et al., 2017). Clusters were only designated when they contained at least two isolates.

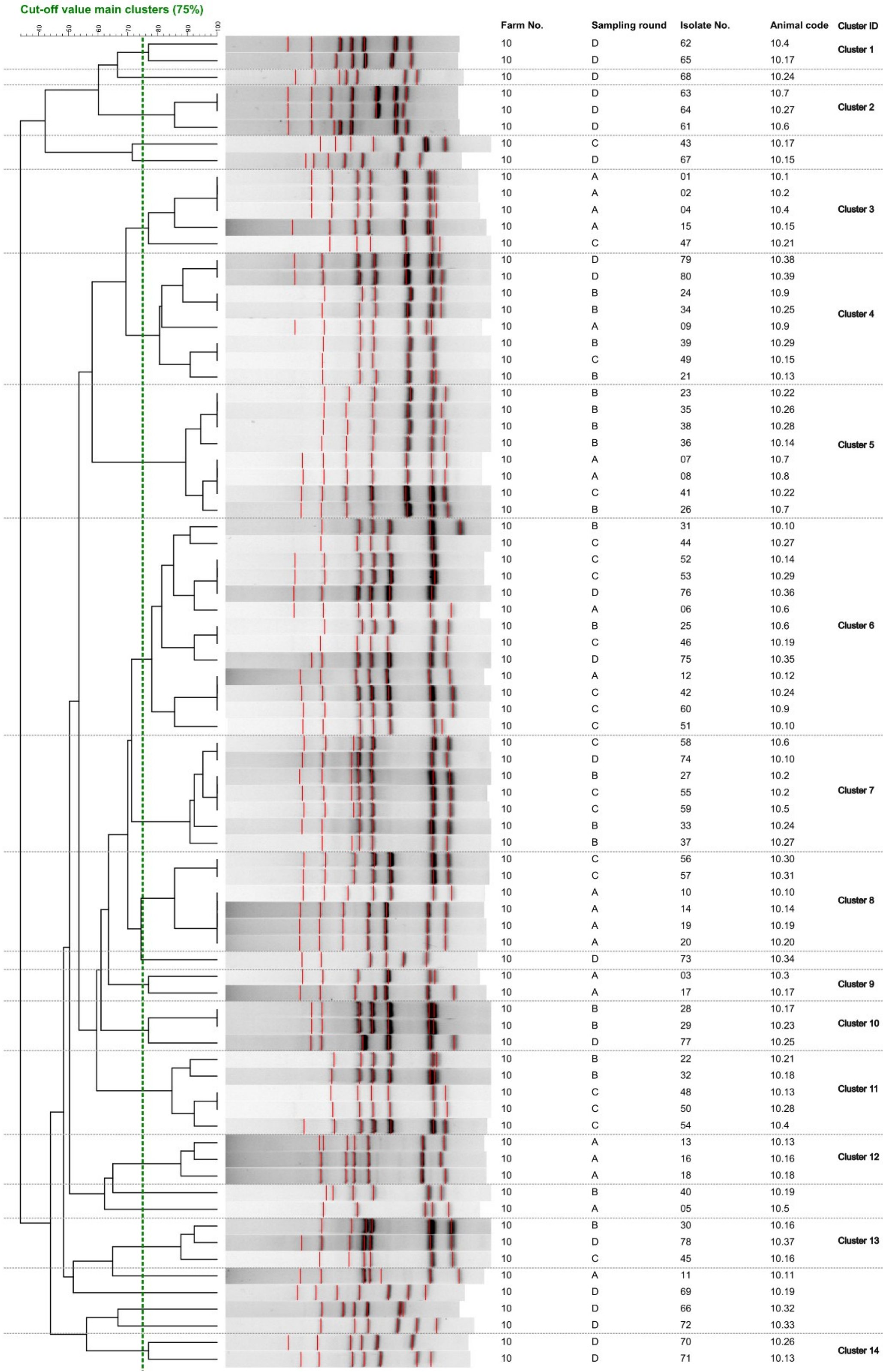

Supplementary Figure 1J. MLVA patterns of 80 *E. coli* isolates recovered on non-selective medium from fecal samples of South American camelids on farm 10 during sampling rounds A–D. Green dotted lines indicate the 75% similarity cutoff used to define clusters (Ranjbar et al., 2017). Clusters were only designated when they contained at least two isolates.

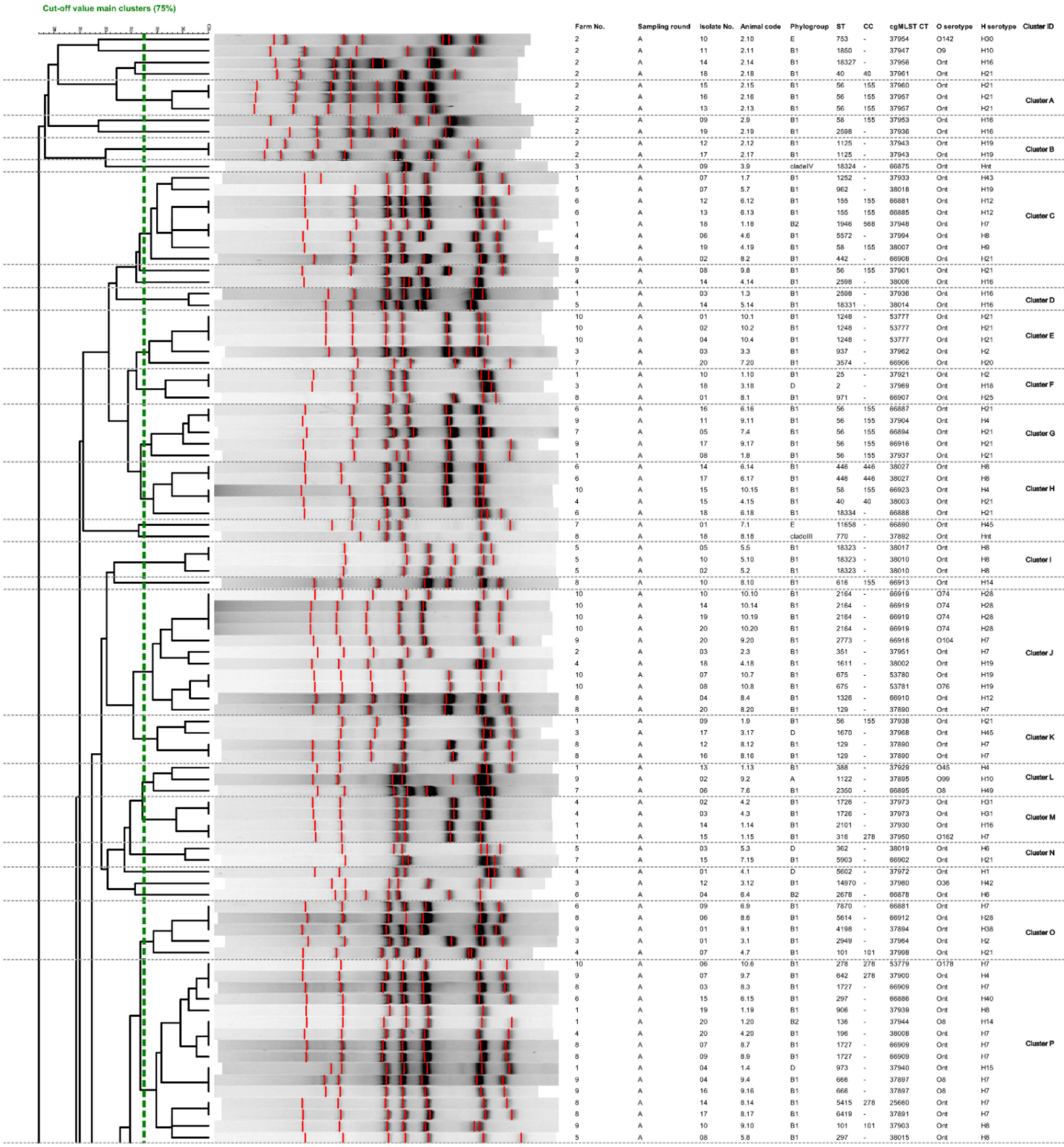

Supplementary Figure 1K. MLVA patterns of 194 *E. coli* isolates recovered on non-selective medium from fecal samples of South American camelids from sampling round A. Strain information (farm number, sampling round, sample number, animal code, phylogroup, sequence type (ST), clonal complex (CC), core-genome multilocus sequence typing complex type (cgMLST CT), and serotype) is indicated on the right. Green dotted lines indicate the 75% similarity cutoff used to define clusters (Ranjbar et al., 2017). Clusters were only designated when they contained at least two isolates.

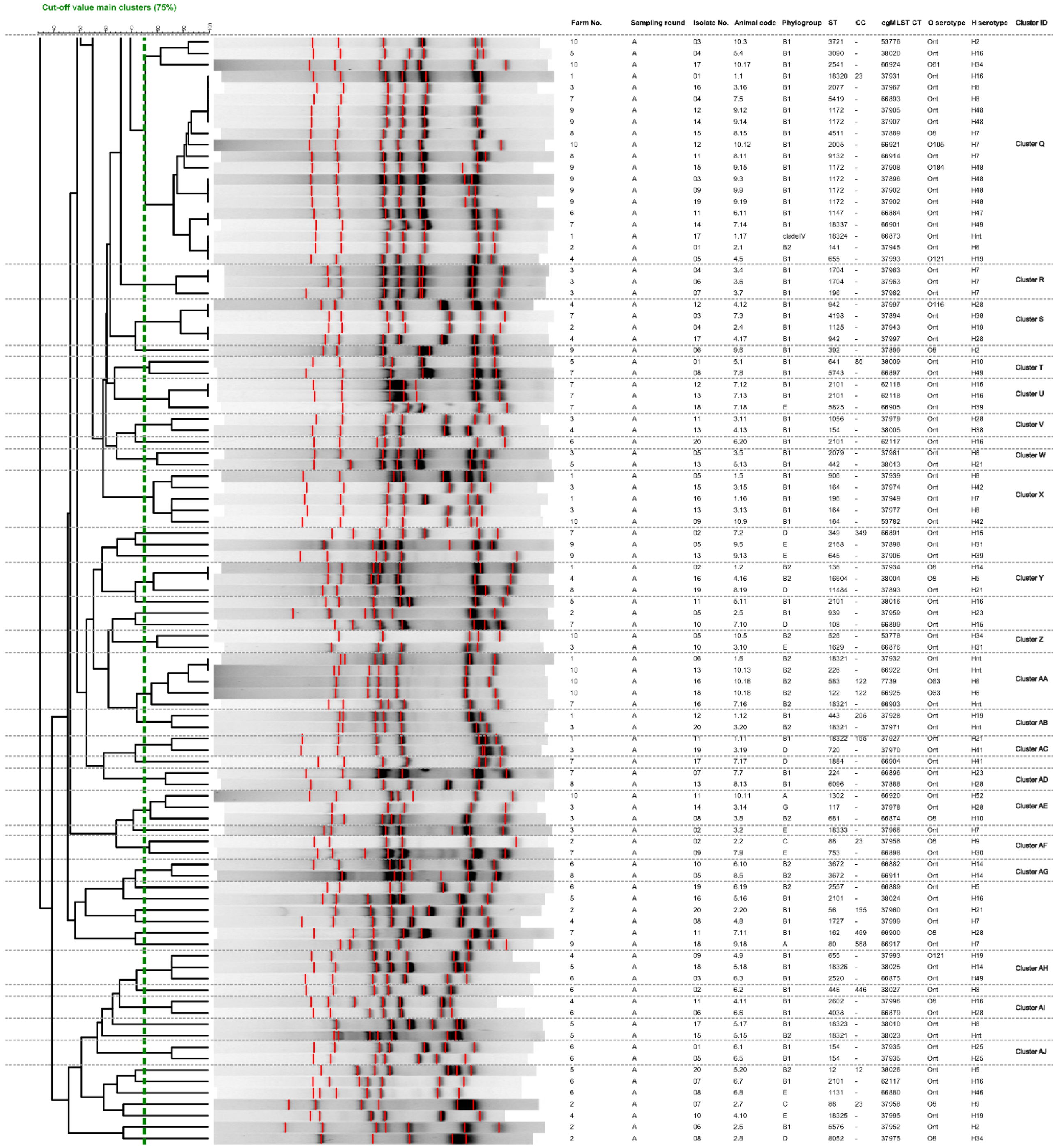

Supplementary Figure 1K (continued). MLVA patterns of 194 *E. coli* isolates recovered on non-selective medium from fecal samples of South American camelids from sampling round A. Strain information (farm number, sampling round, sample number, animal code, phylogroup, sequence type (ST), clonal complex (CC), core-genome multilocus sequence typing complex type (cgMLST CT), and serotype) is indicated on the right. Green dotted lines indicate the 75% similarity cutoff used to define clusters (Ranjbar et al., 2017). Clusters were only designated when they contained at least two isolates.

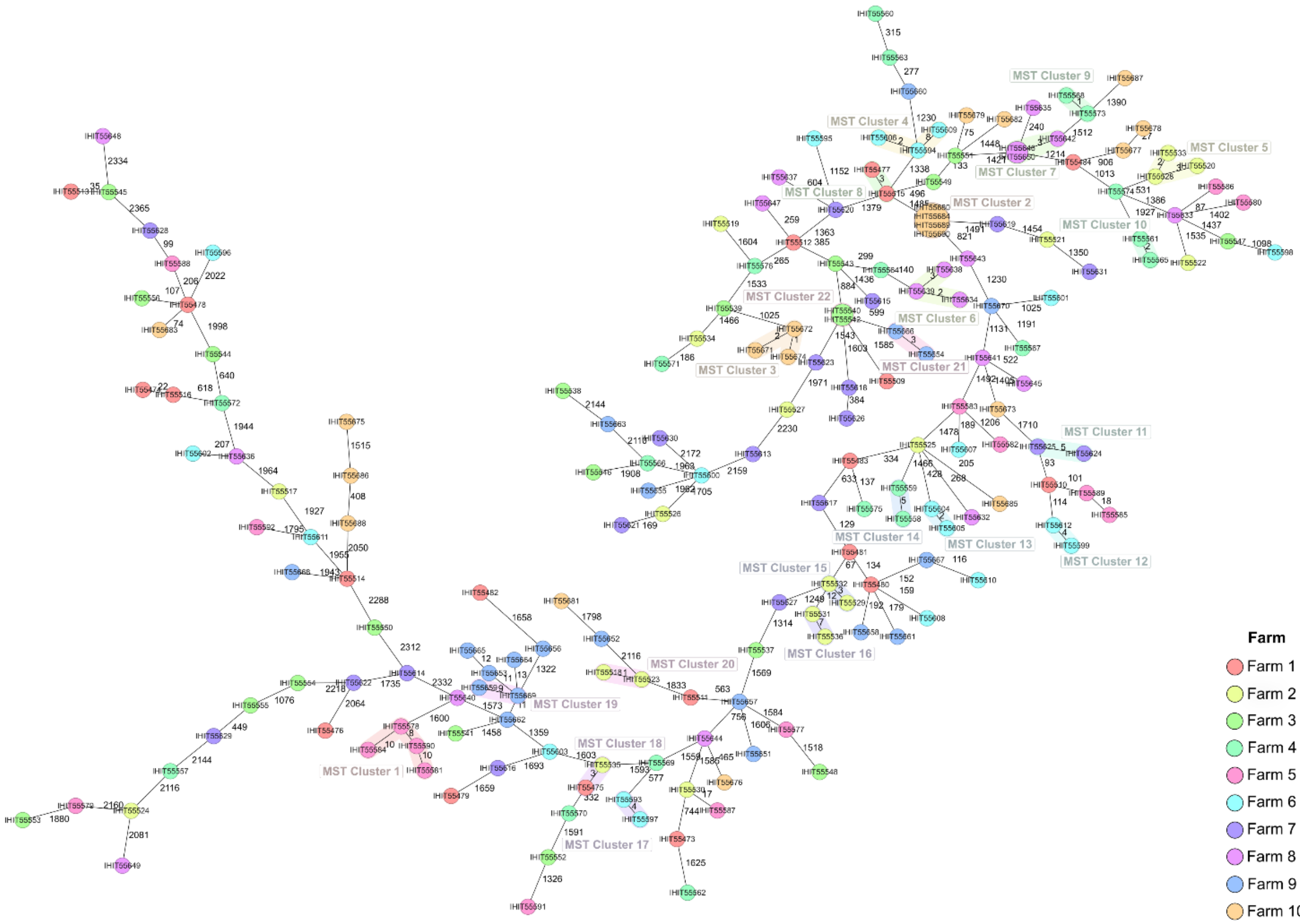

Supplementary Figure 2. Minimum spanning tree (MST) of 194 non-selectively obtained *E. coli* isolates from sampling round A, based on core-genome multilocus sequence typing (cgMLST) allelic distances. Each node represents an individual isolate, and number on the connecting lines indicate allelic differences across the cgMLST scheme. Colors indicate the farm of origin.

1.2 Supplementary Tables

Supplementary Table 1. Number of antimicrobial resistance genes or chromosomal mutations detected in the genomes of 286 *E. coli* isolates obtained by non-selective and selective cultivation

Supplementary Table 2. Genomic characteristics of *E. coli* isolates obtained by non-selective and selective cultivation, including selected InPEC- and ExPEC-associated virulence genes and assigned pathotypes

Supplementary Table 3. Presence of virulence-associated genes in 286 *E. coli* isolates from South American camelids (SACs). Only genes detected in at least one isolate are listed.

Supplementary Table 1. Number of antimicrobial resistance genes or chromosomal mutations detected in the genomes of 286 *E. coli* isolates obtained by non-selective and selective cultivation

No.	Antimicrobial class	Antimicrobial agent	Resistance mechanism	Gene or mutation	Number of positive isolates			
					Non-SEL (n=198)	COL-R (n=18)	CTX-R (n=70)	Total (n=286)
1	Tetracycline	Tetracycline	Acquired resistance gene	*tet*(A)	4	18	10	32
1	Tetracycline	Tetracycline	Acquired resistance gene	*tet*(B)	2	0	1	3
1	Tetracycline	Tetracycline	Acquired resistance gene	*tet*(M)	1	0	33	34
2	Aminoglycoside	Streptomycin	Acquired resistance gene	*aadA1*	5	0	34	39
2	Aminoglycoside	Streptomycin	Acquired resistance gene	*aadA2*	1	0	10	11
2	Aminoglycoside	Streptomycin	Acquired resistance gene	*aadA5*	0	0	1	1
2	Aminoglycoside	Streptomycin	Acquired resistance gene	*aph(6)-Id*	5	0	47	52
2	Aminoglycoside	Streptomycin	Acquired resistance gene	*aph(3'')-Ib*	5	0	47	52
2	Aminoglycoside	Kanamycin	Acquired resistance gene	*aph(3')-Ia*	5	0	33	38
2	Aminoglycoside	Gentamycin	Acquired resistance gene	*aac(3)-IId*	2	0	33	35
2	Aminoglycoside	Streptomycin	Acquired resistance gene	*sat2*	1	0	0	1
3	Rifampin	Rifampin	Acquired resistance gene	*arr-2*	0	0	1	1
4	Lincosamide	Lincosamide	Acquired resistance gene	*lnu(F)*	2	0	23	25
5	Phenicol	Chloramphenicol	Acquired resistance gene	*cmlA1*	3	0	33	36
5	Phenicol	Chloramphenicol	Acquired resistance gene	*cmlA5*	0	0	1	1
5	Phenicol	Chloramphenicol	Acquired resistance gene	*catA1*	1	0	0	1
5	Phenicol	Chloramphenicol, Florfenicol	Acquired resistance gene	*floR*	1	0	3	4
6	Folate pathway antagonist	Sulfonamide	Acquired resistance gene	*sul1*	1	0	4	5
6	Folate pathway antagonist	Sulfonamide	Acquired resistance gene	*sul2*	5	0	20	25
6	Folate pathway antagonist	Sulfonamide	Acquired resistance gene	*sul3*	3	0	27	30
6	Folate pathway antagonist	Trimethoprim	Acquired resistance gene	*dfrA1*	2	0	0	2
6	Folate pathway antagonist	Trimethoprim	Acquired resistance gene	*dfrA5*	2	0	5	7
6	Folate pathway antagonist	Trimethoprim	Acquired resistance gene	*dfrA7*	0	0	1	1
6	Folate pathway antagonist	Trimethoprim	Acquired resistance gene	*dfrA12*	3	0	33	36
6	Folate pathway antagonist	Trimethoprim	Acquired resistance gene	*dfrA14*	0	0	8	8
6	Folate pathway antagonist	Trimethoprim	Acquired resistance gene	*dfrA17*	0	0	5	5
6	Folate pathway antagonist	Trimethoprim	Acquired resistance gene	*dfrA36*	1	0	0	1
6	Folate pathway antagonist	Trimethoprim	Acquired resistance gene	*dfrA51*	0	0	2	2
7	Macrolides	Azithromycin, Erythromycin, Spiramycin, Telithromycin	Acquired resistance gene	*mph(A)*	0	0	11	11
8	Phosphonic antibiotics	Fosfomycin	Acquired resistance gene	*fosA7.5*	2	0	1	3
8	Phosphonic antibiotics	Fosfomycin	Chromosomal mutation	GlpT E448K [a]	196	18	23	237
8	Phosphonic antibiotics	Fosfomycin	Chromosomal mutation	UhpT E350Q [a]	28	0	9	37
8	Phosphonic antibiotics	Fosmidomycin	Chromosomal mutation	CyaA S352T [a]	11	0	6	17
9	Fluoroquinolone	Fluoroquinolone	Acquired resistance gene	*qnrS1*	1	18	8	27
9	Fluoroquinolone	Fluoroquinolone	Acquired resistance gene	*qnrB4*	0	0	4	4
9	Fluoroquinolone	Fluoroquinolone	Acquired resistance gene	*qnrB19*	0	0	5	5
9	Fluoroquinolone	Fluoroquinolone	Chromosomal mutation	GyrA S83L [b]	4	18	37	59
9	Fluoroquinolone	Fluoroquinolone	Chromosomal mutation	GyrA D87N [b]	3	0	36	39
9	Fluoroquinolone	Fluoroquinolone	Chromosomal mutation	ParC S80I [b]	3	0	36	39
9	Fluoroquinolone	Fluoroquinolone	Chromosomal mutation	ParC S57T [b]	3	0	0	3
9	Fluoroquinolone	Fluoroquinolone	Chromosomal mutation	ParE D475E [b]	7	0	0	7
9	Fluoroquinolone	Fluoroquinolone	Chromosomal mutation	ParE S458A [b]	1	0	4	5
9	Fluoroquinolone	Fluoroquinolone	Chromosomal mutation	ParE I355T [b]	3	0	0	3
9	Fluoroquinolone	Fluoroquinolone	Chromosomal mutation	ParE L416F [b]	0	0	3	3
10	Polymyxins	Colistin	Acquired resistance gene	*mcr-1.1*	1	18	0	19
10	Polymyxins	Colistin	Chromosomal mutation	PmrB Y358N [c]	146	0	12	158
10	Polymyxins	Colistin	Chromosomal mutation	PmrB E123D [c]	20	0	3	23

Supplementary Table 1 (continued).

No.	Antimicrobial class	Antimicrobial agent	Resistance mechanism	Gene or mutation	Number of positive isolates			
					Non-SEL (n=198)	COL-R (n=18)	CTX-R (n=70)	Total (n=286)
11	Beta-lactam	Class A β-lactamases	Acquired resistance gene (non-ESBL)	*bla*$_{TEM-1}$	7	0	40	47
				bla$_{TEM-135}$	0	17	0	17
			Acquired resistance gene (ESBL)	*bla*$_{CTX-M-1}$	0	0	7	7
				bla$_{CTX-M-14}$	0	0	2	2
				bla$_{CTX-M-15}$	0	0	9	9
				bla$_{CTX-M-27}$	0	0	1	1
				bla$_{CTX-M-55}$	2	0	33	35
		Class C β-lactamases	Acquired resistance gene (AmpC β-lactamase)	*bla*$_{DHA-1}$	0	0	4	4
				bla$_{CMY-2}$	0	0	4	4
				bla$_{EC-5}$	2	0	0	2
			Chromosomal mutation	*ampC* C42T	0	0	2	2
		Class D β-lactamases	Acquired resistance gene	*bla*$_{OXA-1}$	1	0	0	1
				bla$_{OXA-10}$	0	0	1	1

[a] The mutations are associated with fosfomycin and fosmidomycin resistance (Moghnia and Al-Sweih, 2022; Fang et al., 2023).
[b] The fluoroquinolone- associated mutations are confirmed to confer fluoroquinolone resistance (Komp Lindgren et al., 2003; Vianello et al., 2021; Chekole et al., 2025).
[c] Mutations in PmrB (Y358N and E123D) are currently not considered colistin resistance determinants (Butters et al., 2024) and are presented here for completeness only. Phenotypic colistin susceptibility testing of non-SEL isolates revealed no resistance phenotype, whereas CTX-R isolates carrying these mutations were not phenotypically tested for colistin resistance.

Supplementary Table 2. Genomic characteristics of *E. coli* isolates obtained by non-selective and selective cultivation, including selected InPEC- and ExPEC- associated virulence genes and assigned pathotypes

Farm No.	Sample ID	Isolation media	Selected virulence-associated genes*	InPEC/ ExPEC	Pathotype	MLST	cgMLST complex type	Phylo-group
1	IHIT55482	Non-SEL	*stx1c, stx2b*	InPEC	STEC	ST25	37921	B1
	IHIT60213	CTX	*cnf1, focACFGI, hlyABCD, papBCDFHIJKX, sfaBCDEGX*	ExPEC	UPEC	ST75	67904	B1
2	IHIT55517	Non-SEL	*focCDFI, hlyABCD, papBCDFHIJK, sfaBDEFGHSY*	ExPEC	UPEC	ST141	37945	B2
4	IHIT60146	CTX	*eae*	InPEC	AEEC	ST589	67905	B2
	IHIT60150	CTX	*eae*	InPEC	AEEC	ST589	67905	B2
	IHIT60151	CTX	*eae*	InPEC	AEEC	ST589	67905	B2
5	IHIT55585	Non-SEL	*stx2b*	InPEC	STEC	ST2101	38016	B1
	IHIT55589	Non-SEL	*stx2b*	InPEC	STEC	ST2101	38024	B1
	IHIT55592	Non-SEL	*focCDF, hlyABD, papBCDFIJKX, sfaBCEFGHXY*	ExPEC	UPEC	ST12	38026	B2
6	IHIT55596	Non-SEL	*eae*	InPEC	AEEC	ST2678	66878	B2
	IHIT55599	Non-SEL	*stx2b*	InPEC	STEC	ST2101	62117	B1
	IHIT55610	Non-SEL	*stx2d*	InPEC	STEC	ST18334	66888	B1
	IHIT55612	Non-SEL	*stx2b*	InPEC	STEC	ST2101	62117	B1
7	IHIT55617	Non-SEL	*stx2b*	InPEC	STEC	ST56	66894	B1
	IHIT55624	Non-SEL	*stx2b*	InPEC	STEC	ST2101	62118	B1
	IHIT55625	Non-SEL	*stx2b*	InPEC	STEC	ST2101	62118	B1
	IHIT55628	Non-SEL	*stx2b*	InPEC	STEC	ST18321	66903	B2
8	IHIT55633	Non-SEL	*stx2b, stx2d*	InPEC	STEC	ST442	66908	B1
9	IHIT55667	Non-SEL	*stx2d*	InPEC	STEC	ST56	66916	B1
10	IHIT55675	Non-SEL	*eae*	InPEC	AEEC	ST526	53778	B2
	IHIT55676	Non-SEL	*stx1c*	InPEC	STEC	ST278	53779	B1
	IHIT55677	Non-SEL	*stx1c, stx2b*	InPEC	STEC	ST675	53780	B1
	IHIT55678	Non-SEL	*stx1c, stx2b*	InPEC	STEC	ST675	53781	B1
	IHIT55685	Non-SEL	*afaABCDEVIII, afaF-VII, cdtABC, iutA*	ExPEC	SEPEC	ST58	66923	B1
	IHIT55686	Non-SEL	*stx2f, eae*	InPEC	AEEC, STEC	ST583	7739	B2
	IHIT55688	Non-SEL	*eae*	InPEC	AEEC	ST122	66925	B2

* Only virulence genes detected that are relevant for the prediction of pathotypes are listed; additional virulence genes identified in the isolates are summarized in Supplementary Table 3). InPEC = positive for at least one of the following virulence factors (and encoding genes): F4 (*faeG*), F5 (*fanA*), F6 (*fasA*), F18 (*fedA*) and F41 (*fimF41a*) (adhesive fimbriae); LT-Ia, LT-Ib (*eltB-Ip*), ST-Ia (*estap*) and ST-II (*estb*) (heat-labile/stable enterotoxins); *stx1* and/or *stx2* (Shiga toxin) or intimin (*eae*) (Göpel et al., 2024). Pathotypes: AEEC, positive for (*eae*); STEC, positive for (*stx1* and/or *stx2*); (Bugarel et al., 2011). ExPEC if positive for ≥2 of five key markers: *papA* and/or *papC* (P fimbriae), *sfa/foc* (S and F1C fimbriae), *afa/dra* (Dr-binding adhesins), *iutA* (aerobactin system), and *kpsMT* II (Johnson et al., 2003; Sarowska et al., 2019); UPEC = ExPEC plus *cnf1* or *hlyD* (Toxins) (Aurich et al., 2023); SEPEC = ExPEC plus one of these genes *cnf1, cnf2, cdtB, hlyF* (Toxins) (Mitchell et al., 2015; Sarowska et al., 2019), SEPEC is heterogeneous, its genes overlap with APEC and UPEC, and it has no single defining molecular profile (Ananias and Yano, 2008).

REFERENCES

Ananias, M., and Yano, T. (2008). Serogroups and virulence genotypes of *Escherichia coli* isolated from patients with sepsis. *Braz J Med Biol Res* 41, 877–883. doi: 10.1590/S0100-879X2008001000008

Aurich, S., Wolf, S. A., Prenger-Berninghoff, E., Thrukonda, L., Semmler, T., and Ewers, C. (2023). Genotypic Characterization of Uropathogenic *Escherichia coli* from Companion Animals: Predominance of ST372 in Dogs and Human-Related ST73 in Cats. *Antibiotics (Basel)* 13. doi: 10.3390/antibiotics13010038

Bugarel, M., Martin, A., Fach, P., and Beutin, L. (2011). Virulence gene profiling of enterohemorrhagic (EHEC) and enteropathogenic (EPEC) *Escherichia coli* strains: a basis for molecular risk assessment of typical and atypical EPEC strains. *BMC Microbiol* 11. doi: 10.1186/1471-2180-11-142

Butters, A., Jovel, J., Gow, S., Liljebjelke, K., Waldner, C., and Checkley, S. L. (2024). PmrB Y358N, E123D amino acid substitutions are not associated with colistin resistance but with phylogeny in *Escherichia coli*. *Microbiol Spectr* 12. doi: 10.1128/spectrum.00532-24

Chekole, W. S., Potgieter, L., Adamu, H., Sternberg-Lewerin, S., Tessema, T. S., and Magnusson, U. (2025). Genomic insights into antimicrobial resistance and virulence of *E. coli* in central Ethiopia: a one health approach. *Front Microbiol* 16, 1597580. doi: 10.3389/fmicb.2025.1597580

Fang, Y., Tran, F., Stanford, K., and Yang, X. (2023). Stress Resistance and Virulence Gene Profiles Associated with Phylogeny and Phenotypes of *Escherichia coli* from Cattle. *J Food Prot* 86, 100122. doi: 10.1016/j.jfp.2023.100122

Göpel, L., Prenger-Berninghoff, E., Wolf, S. A., Semmler, T., Bauerfeind, R., and Ewers, C. (2024). Repeated Occurrence of Mobile Colistin Resistance Gene-Carrying Plasmids in Pathogenic *Escherichia coli* from German Pig Farms. *Microorganisms* 12. doi: 10.3390/microorganisms12040729

Johnson, J. R., Gajewski, A., Lesse, A. J., and Russo, T. A. (2003). Extraintestinal pathogenic *Escherichia coli* as a cause of invasive nonurinary infections. *J Clin Microbiol* 41, 5798–5802. doi: 10.1128/JCM.41.12.5798–5802.2003

Komp Lindgren, P., Karlsson, A., and Hughes, D. (2003). Mutation rate and evolution of fluoroquinolone resistance in *Escherichia coli* isolates from patients with urinary tract infections. *Antimicrob Agents Chemother* 47, 3222–3232. doi: 10.1128/AAC.47.10.3222–3232.2003

Mitchell, N. M., Johnson, J. R., Johnston, B., Curtiss, R., and Mellata, M. (2015). Zoonotic potential of *Escherichia coli* isolates from retail chicken meat products and eggs. *Appl Environ Microbiol* 81, 1177–1187. doi: 10.1128/AEM.03524-14.

Moghnia, O. H., and Al-Sweih, N. A. (2022). Whole Genome Sequence Analysis of Multidrug Resistant *Escherichia coli* and *Klebsiella pneumoniae* Strains in Kuwait. *Microorganisms* 10. doi: 10.3390/microorganisms10030507

Ranjbar, R., Pezeshknejad, P., Khamesipour, F., Amini, K., and Kheiri, R. (2017). Genomic fingerprints of *Escherichia coli* strains isolated from surface water in Alborz province, Iran 10, 295. doi: 10.1186/s13104-017-2575-z

Sarowska, J., Futoma-Koloch, B., Jama-Kmiecik, A., Frej-Madrzak, M., Ksiazczyk, M., Bugla-Ploskonska, G., et al. (2019). Virulence factors, prevalence and potential transmission of extraintestinal pathogenic *Escherichia coli* isolated from different sources: recent reports. *Gut Pathog* 11. doi: 10.1186/s13099-019-0290-0

Vianello, M. A., Cardoso, B., Fuentes-Castillo, D., Moura, Q., Esposito, F., Fuga, B., et al. (2021). International high-risk clone of fluoroquinolone-resistant *Escherichia coli* O15:H1-D-ST393 in remote communities of Brazilian Amazon. *Infect Genet Evol* 91, 104808. doi: 10.1016/j.meegid.2021.104808
